# Overview of Membrane Protein Sample Preparation for Single-Particle Cryo-Electron Microscopy Analysis

**DOI:** 10.3390/ijms241914785

**Published:** 2023-09-30

**Authors:** Catherine Vénien-Bryan, Carlos A. H. Fernandes

**Affiliations:** Unité Mixte de Recherche (UMR) 7590, Centre National de la Recherche Scientifique (CNRS), Muséum National d’Histoire Naturelle, Institut de Recherche pour le Développement (IRD), Institut de Minéralogie, Physique des Matériaux et de Cosmochimie (IMPMC), Sorbonne Université, 75005 Paris, France; catherine.venien-bryan@sorbonne-universite.fr

**Keywords:** membrane proteins, cryo-electron microscopy, detergents, nanodiscs, amphipols, structural biology

## Abstract

Single-particle cryo-electron microscopy (cryo-EM SPA) has recently emerged as an exceptionally well-suited technique for determining the structure of membrane proteins (MPs). Indeed, in recent years, huge increase in the number of MPs solved via cryo-EM SPA at a resolution better than 3.0 Å in the Protein Data Bank (PDB) has been observed. However, sample preparation remains a significant challenge in the field. Here, we evaluated the MPs solved using cryo-EM SPA deposited in the PDB in the last two years at a resolution below 3.0 Å. The most critical parameters for sample preparation are as follows: (i) the surfactant used for protein extraction from the membrane, (ii) the surfactant, amphiphiles, nanodiscs or other molecules present in the vitrification step, (iii) the vitrification method employed, and (iv) the type of grids used. The aim is not to provide a definitive answer on the optimal sample conditions for cryo-EM SPA of MPs but rather assess the current trends in the MP structural biology community towards obtaining high-resolution cryo-EM structures.

## 1. Introduction

Membrane proteins (MPs) play a crucial role in various essential functions in the cells, including the regulation of molecule transport across the membrane, cell–cell recognition, signal transduction, cell adhesion, and control of membrane lipid composition [1]. MPs, when dysfunctional, are also involved in several diseases, including cancers, viral infections, channelopathies and neurodegenerative disorders, being the target of more than 50% of currently marketed drugs [2,3]. The obtention of high-resolution structures of MPs is essential to a comprehensive understanding of their mechanisms of action and for the successful design of new drugs [4].

Over the past three decades, X-ray crystallography has been the prevailing technique for determining high-resolution structures of MPs, as well as other macromolecules and complexes. However, this technique faces a significant challenge in obtaining well-ordered 3D crystals, which require large amounts of samples. This challenge is particularly pronounced for MPs due to their low yield per liter of cell culture in heterologous expression systems [1]. Furthermore, the surfactants employed to extract MPs from the host cell membrane have a detrimental effect on protein stability, impairing the crystallization process [5].

**Figure 1:**
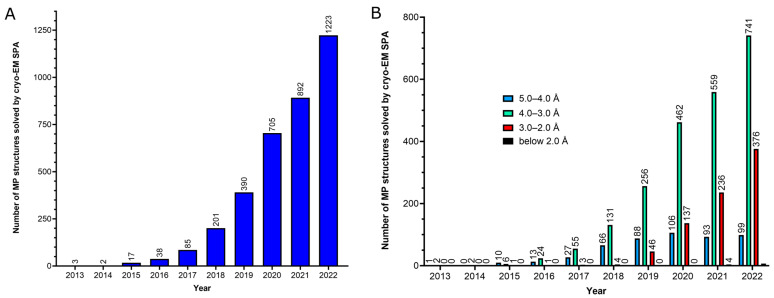
The number of structures of membrane proteins deposited in the Protein Data Bank (PDB) solved using cryo-EM single-particle analysis since the first atomic structure of a membrane protein was determined using this technique in 2013. (**A**) Number of membrane protein structure deposits per year; (**B**) number of membrane protein structure deposits per year and per resolution range.

In this context, single-particle cryo-electron microscopy (cryo-EM SPA) has emerged as an exceptionally suitable technique for determining the structure of MPs. Unlike X-ray crystallography, cryo-EM SPA eliminates the need for crystal formation, requires much smaller sample quantities, and allows the use of a wide variety of hydrophobic environments [6,7]. The impact of cryo-EM SPA on the field of studying the structural biology of MPs can be illustrated by some numbers since the first atomic structures of a MP were determined through the use of this technique in 2013 [8,9]. While the use of X-ray crystallography has yielded a total of 5,828 deposits of MP structures in the Protein Data Bank (PDB) in all history, cryo-EM SPA has led to 3,556 deposits of MP structures in just the last ten years (2013–2022). Notably, this huge progress has been concentrated in the last four years (2019–2022; Figure 1A), which account for 90% of the total number of cryo-EM SPA deposits of MP structures. Furthermore, there has been a remarkable increase in MP structures solved through the use of cryo-EM SPA at better than 3.0 Å resolution in the last three years (a 174% increase in 2022 compared to 2020; Figure 1B), with 11 structures reaching better than 2.0 Å resolution appearing in the last two years.

Cryo-EM SPA is a technique that involves the application of purified macromolecules onto cryo-EM grids, which are then rapidly frozen to embed the molecules in a thin layer of vitreous ice (vitrification step). The ice layer thickness must be thin enough to support all orientations of the vitrified molecule, as thicker layers can lead to a significant increase in noise in the high-resolution information [10]. Then, the cryo-EM grid is transferred to a transmission electron microscope (TEM), where images are acquired under cryogenic conditions [6,11]. The image processing pipeline to obtain the final 3D cryo-EM map of a target molecule encompasses several steps [12]. Briefly, each captured image consists of a movie composed of dose-fractionated frames, which require motion estimation and correction. The corrected images, known as micrographs, are then used to estimate the contrast transfer function (CTF) parameters, which describe how aberrations in the TEM affect the image of the samples. Additionally, micrographs are utilized to locate and select single particles. The particles are extracted from the micrographs and subjected to iterative classifications to sort out suitable ones for constructing the 3D cryo-EM map. Finally, the atomic structure of the molecule is built within the 3D cryo-EM map, resulting in the final structure of the target molecule [12].

The significant increase in the popularity of cryo-EM SPA among structural biologists in the last ten years is a result of several technical advancements, often referred to as the ‘resolution revolution’ [13]. One of the most significant technical advancements was the introduction of complementary metal–oxide–semiconductor (CMOS) direct detector device (DDD) cameras, which improved the detective quantum efficiency (DQE) compared to charged-coupled device (CCD) and other detectors [14]. Moreover, DDD cameras have a high frame rate that allows the data to be captured as dose-fractionated image stacks rather than a single micrograph. This approach enables the correction of motion induced by stage or beam movements, leading to a substantial improvement in image quality by recovering the high-resolution signal that may have been compromised by such motion [15,16]. Moreover, data processing has become more robust and user-friendly with automated pipelines in different software [17,18,19]. Other technical advances continue to be continuously made in recent years, which has allowed high-resolution data to be obtained more and more routinely. Notably, the new generation of field emission fun (cold FEG) and the introduction of post-column imaging filters, such as Selectris and Selectris X, has significantly enhanced the signal-to-noise ratio (SNR) and contrast of TEM images [20]. Additionally, the latest generation of DDD cameras (Falcon 4) enables the acquisition of images with super-resolution information at a significantly faster rate, up to ten times faster, while also producing smaller file sizes [20,21].

In view of these great advancements, sample preparation arises as the most important challenge for cryo-EM SPA, especially for MPs, which require their extraction and purification from the membrane and their stabilization in an aqueous environment. This is typically achieved by solubilizing the MPs in surfactants that can be later replaced with more suitable surfactant or amphiphile molecules prior to the vitrification step. A recent quantitative analysis examined the use of surfactants and other types of molecules employed for protein extraction from the membrane and during the vitrification step in all unique cryo-EM structures of MPs solved up to 2020 [22]. However, at that time, only 192 MP structures were available at near-atomic resolution (better than 3.0 Å resolution). However, in the last two years (2021–2022), new 623 MP structures have been released at this resolution level (more than three times the number of structures released up to the year 2020 with ≤3.0 resolution) (Figure 1B). Thus, in light of the significant increase in the number of MP structures solved by using cryo-EM SPA at very high resolution in the last two years, we present an updated and expanded analysis of the surfactants and other molecules used for protein extraction and at the vitrification step, focused solely on MP structures solved at near-atomic resolution. Additionally, we assessed the vitrification method and the type of grids employed for obtaining these structures. Our objective is not to provide a definitive answer regarding the perfect sample conditions for cryo-EM SPA for MPs but rather to evaluate the current trends in the MP structural biology community towards obtaining cryo-EM structures at resolutions better than 3.0 Å.

To conduct this analysis, we searched the Protein Data Bank (PDB) for deposited structures of MPs (annotated as PDBTM, MemPROTMD, OPM or mpstruc in PDB) solved by using cryo-EM SPA at a resolution better than 3.0 Å in the last two years (2021–2022). We then examined the published manuscripts referring to these structures for the following sample preparation parameters: (i) the surfactant used for MP extraction from the membrane, (ii) the surfactant, amphiphiles, nanodiscs or other molecules present in the vitrification step, (iii) the vitrification method employed, and (iv) the type of grids used. It is worth noting that some manuscripts may include multiple structures obtained under different states (e.g., open and closed conformations, presence of different ligands, and others). To avoid bias of more extensively studied proteins and provide greater representativeness of all MPs structural molecular biological groups, a single structure from each manuscript was analyzed unless one of the parameters analyzed above was different (unique reports). In a singular instance, we considered two structures from a single manuscript because it presented two entirely distinct high-resolution MP structures [23]. As a result, we analyzed a total of 302 different manuscripts, resulting in 303 unique reports of MP structures, 306 unique reports analyzed for the surfactant used for MP extraction, 312 unique reports analyzed for the surfactant present in the vitrification step, 303 unique reports analyzed for the vitrification method employed, and 310 unique reports analyzed for the type of grids employed. A list containing all the 302 manuscripts analyzed here is available in the Supplementary Information.

## 2. Functional Analysis of the MP Structural Reports

**Figure 2:**
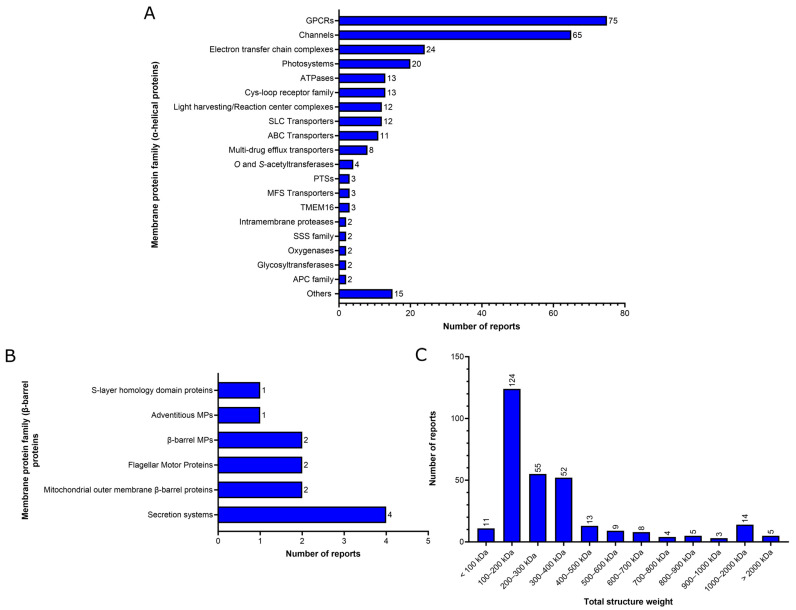
Family and size total structure weight distribution of the unique reports of membrane proteins whose structure has been solved at a resolution better than 3 Å by cryo-EM SPA during the last two years (2021–2022) available in the Protein Data Bank. (**A**) Family groups of α-helical MPs. Other include a single report for the following families: Adventitious MPs/α -helical pore-forming toxins; Bacterial rhodopsin; Wntless (WLS Transporters); Chain length determinant and associated proteins; Sterol-sensing domain (SSD) proteins; Energy-coupling factor (ECF) transporters; Host-defense proteins; Cellulose synthase; Autoinducer exporters; Amino acid secondary transporters; Yellow stripe 1 transporter; Sec, translocase, and insertase proteins; Antiporters; Cysteine proteases; and PIN-FORMED (PIN) proteins. (**B**) Family groups of β-barrel MPs. β-barrel MPs family includes one report of porins and relatives and one of monomeric/dimeric. Adventitious MPs included a report of β-sheet pore-forming toxins/attack complexes. (**C**) Total structure weight distribution of all unique reports of membrane proteins identified in this work. Total structure weight comprises the molecular weight of all non-water atoms in the PDB deposited model. The family groups were classified according to the family name provided by the mpstruc database (https://blanco.biomol.uci.edu/mpstruc/ accessed on 14 August 2023). GPCRs: G-protein coupled receptors; SLC: solute carrier; ABC: ATP-binding cassette; PTS: phosphoenolpyruvate-dependent phosphotransferase: MFS: major facilitator superfamily; SSS: solute sodium symporter; APC: Amino Acid/Polyamine/Organocation.

Prior to delving into the analysis of the sample preparation parameters for cryo-EM SPA of MPs, we performed a functional analysis of the 303 unique reports of MP structures solved by using cryo-EM SPA at a resolution better than 3.0 Å in the last two years (2021–2022). This analysis revealed that 291 of the 303 unique reports (96%) of MP structures were from α-helical MPs, in contrast to only 12 of the 303 unique reports (4%) from β-barrel MPs (Figure 2A,B). Any high-resolution structure of monotopic MP was identified in this study. Regarding α-helical MPs, the most abundant high-resolution MP structures identified here were G-protein coupled receptors (GPCRs) (75 out of 303 reports, 25%) and channels (65 out of 303 reports, 21%) (Figure 2A). We also provided an analysis of the total structure weight of the unique reports of MP structures identified (Figure 2C). The total structure weight comprises the molecular weight of all the non-water atoms in the PDB deposited model. It is important to note that the total structure weight also comprises the mass of any other proteins contained in the deposit, such as Fab fragments, antibodies, or nanobodies. These additional binding partners are often used for complex stabilization, particularly in the case of GPCRs, or to augment the size of the protein, enabling the analysis of small proteins by cryo-EM SPA (more discussion about this topic in Section 3.2). The most frequent total structure weight range identified here was 100 to 200 kDa (124 of 303 reports, 40%) (Figure 2C), which contains 64 out of 75 GPCRs reports and only 7 out of 65 channels reports. Unlike GPCR reports, which are the majority located in the 100–200 kDa range, channels are present in reports across almost all total structure weight ranges categorized here (Figure 2C), with the exception of only the 700–800 kDa range. The most commonly found total structure weight ranges for channels are 300–400 kDa (22 out of 65 channel reports) and 200–300 kDa (17 out of 65 channel reports). It is important to note that from the 11 reported structures with a total structure weight smaller than 100 kDa, 7 of them did not use any additional binding partners to increase the protein size, including the report of the smallest MP identified here (voltage-dependent chloride channel VCNN1 reconstituted in nanodiscs at 2.7 Å resolution), with a total structure weight of 42 kDa (PDB ID 7EK2) [24]. Photosystems are the majority in terms of total structure weight ranges greater than 1000 kDa (7 out of 19 reports).

## 3. Sample Preparation

### 3.1. Amphiphiles Used for Membrane Protein Extraction

MPs are characterized by their dual hydrophobic–hydrophilic nature, which arises from the presence of a highly hydrophobic transmembrane domain, along with an hydrophilic extracellular and cytoplasmic domains within their structures. Consequently, maintaining MPs in a soluble and stable state outside the lipid biological bilayer poses a considerable challenge, as they have a heightened tendency to aggregate or denature in an aqueous medium [25]. Therefore, upon the expression of MPs in heterologous systems, it becomes necessary to extract them from the lipid bilayer and replace it with another amphiphilic system for subsequent structural and functional studies. Various surfactants have been developed to provide a suitable environment for MP extraction from the membrane, with detergents being the historically first ones used for this purpose [26]. Detergents are amphipathic molecules consisting of a hydrophilic head group (typically polar, sometimes charged) and a lipophilic or hydrophobic (apolar) tail. This architecture allows detergents to insert their lipophilic tails into the lipidic membrane, thereby disrupting it and extracting the MP from the membrane as the detergent concentration increases. As a result of the hydrophilic–lipophilic balance of detergent molecules, they form spontaneously micelles, which are pseudo-spherical assemblies. Micelle formation occurs once the critical micelle concentration (CMC) is reached and when the sample is above critical micellar temperature. Through micelle formation, an MP becomes part of the detergent–protein complex, sometimes leading to the complete loss of surrounding lipids [27].

**Figure 3:**
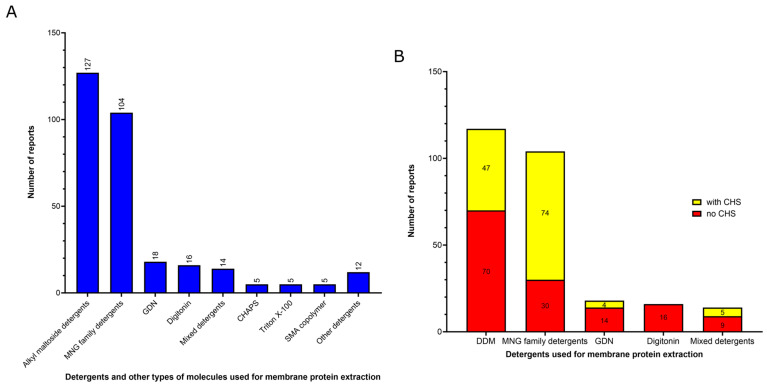
Profile of detergents and other types of molecules usage during the membrane extraction step of the membrane proteins whose structure has been solved at a resolution better than 3 Å by cryo-EM SPA during the last two years (2021–2022) available in the Protein Data Bank. (**A**) Number of reports found for each surfactant or other molecules used for the protein membrane extraction. Alkyl maltoside detergents include DDM, DM and UDM. MNG family detergents include LMNG, MNG and DMNG. Mixed detergents include associations between different detergents. In this class, we noticed the reports of the association between DDM and sodium cholate (3 reports), DDM and LMNG (2 reports), DDM and DM-NPG (2 reports), DDM and GDN (1 report), LMNG and digitonin (2 reports), LMNG and GDN (1 report), LMNG and sodium cholate (1 report), digitonin and LDAO (1 report), DDM and Triton X-100 (1 report). Other detergents include DDM analog cyclohexyl-α maltoside (t-PCCαM; 3 reports), Cymal-4 (1 report), Cymal-6 (1 report), LDAO (2 reports), Sodium cholate (1 report), OGNG (1 report), C12E9 (1 report), C12E8 (1 report), and glycerol (1 report). (**B**) Analysis of the addition of cholesteryl hemisuccinate (CHS) on surfactant-solubilized MPs at the five most used detergents used for protein membrane extraction step.

Among the MP structures solved by using cryo-EM SPA at near-atomic resolution (better than 3.0 Å resolution), almost all molecules employed for membrane protein extraction are detergents (301 of 306 reports). The only exceptions were the five cases where styrene–maleic acid copolymer (SMA) was used (Figure 3A). This is a free detergent system where SMA copolymers solubilize MPs directly from the membrane, keeping the native lipid environment from forming polymer-bounded nanodiscs, often called native nanodiscs [28]. This approach addresses the challenge of the transient protein destabilization caused by detergents by dissolving integral membrane proteins from biological membranes into nanosized discs. Within these nanoparticles, proteins are embedded in a patch of their native lipid bilayer, which is stabilized in solution by the amphipathic polymer that envelops the disc [28]. This detergent-free approach for membrane protein solubilization offers significant simplifications in terms of the purification and manipulation of the samples. However, it poses some considerable difficulties for the conjugation of functional groups to the membrane proteins, which is often required for their biochemical and biophysical characterization [29]. Recently, an anhydride form of SMA (SMAnh) has been introduced, capitalizing on the reactivity of maleic anhydride with alcohols and amines. This reactivity has been used to prepare biologically active conjugates of SMA with small molecules such as fluorophores, drugs, and proteins [29].

The four most common detergents used for membrane protein extraction are, in order of frequency of use (Figure 3A):

(1) Detergents with a single maltose-based polar moiety and single alkyl chain (DDM, UDM, DM): 127 of 306 reports (41%). In this group, the most widely used detergent is DDM, which is present in 117 of 127 reports of this detergent class. DM is present in 9 of 131 reports and UDM in 1 of 131 reports;
(2) Detergents belonging to the maltoside–neopentyl glycol (MNG) family (MNG, LMNG and DMNG): 104 of 306 reports (34%). In this group, the most widely used detergent is LMNG, which is present in 101 of 104 reports of this detergent class. MNG is present in 2 of 104 reports and DMNG is present in 1 of 107 reports;
(3) Glyco-diosgenin (GDN): 18 of 306 reports (6%);
(4) Digitonin: 16 of 306 reports (5%).

**Figure 4:**
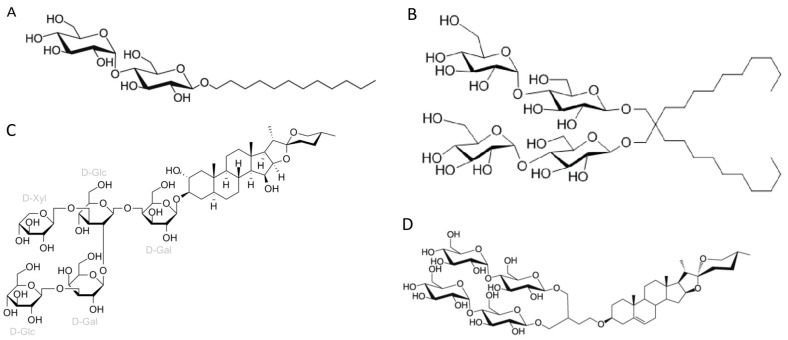
Chemical structures of the four most common detergents used for membrane protein extraction step of the membrane protein structures solved using cryo-EM single-particle analysis in the last two years (2021–2022) available in the Protein Data Bank. (**A**) n-Dodecyl-β-D-maltopyranoside (DDM); (**B**) Lauryl maltose neopentyl glycol (LMNG); (**C**) Digitonin; (**D**) Glyco-diosgenin (GDN).

DDM (n-Dodecyl-β-D-maltopyranoside), as well as their relatives DM (n-Decyl-β-maltoside and UDM (n-Undecyl-β-maltoside), is a detergent that belongs to alkyl maltoside class. DDM was developed in 1980 and has been widely used in X-ray crystallography [27]. As demonstrated here, it is still the preferred detergent for extracting membrane proteins for high-resolution cryo-EM studies. The polar head of DDM is provided by a disaccharide (maltose), while its hydrophobic portion consists of a 12-carbon alkyl chain (Figure 4A). DDM proves to be highly effective for membrane protein extraction, and being non-ionic, it is able to maintain the stable native state of numerous proteins. Additionally, DDM exhibits a low CMC value (0.17 mM), which allows reduced detergent usage. It forms well-defined micelles, albeit relatively large in size and surrounds protein with a significant and mobile belt-like structure [26].

LMNG (lauryl maltose neopentyl glycol) is a detergent that belongs to the maltoside–neopentyl glycol (MNG) family. This detergent class is characterized as having a maltoside head group and two alkyl chains and two carbohydrate head groups connected with each other via a neopentyl glycol unit in the central region, which result in a dimeric architecture compared with detergents from alkyl maltoside class. LMNG, for example, is a dimer of DDM, thus presenting two maltoside units in their hydrophilic domain and two alkyl chains (Figure 4B). This molecular rearrangement of LMNG results in a CMC value even lower than DDM (0.01 mM rather than 0.17 mM), although it has been reported to form larger micelles [26,30,31].

Although DDM is still the most widely used detergent for membrane protein extraction, even for cryo-EM studies, its once clear dominance seems to have slightly decreased in recent years. In comparison to a previous report [22], our analysis focusing on high-resolution cryo-EM structures, indicates a slight decrease in DDM usage from membrane protein extraction (~41% compared to ~43% from the previous report) and a significant rise in the utilization of detergents from the MNG family, particularly LMNG (~34% compared to ~22% from the previous report). Originally employed with remarkable success for the solubilization and stabilization of GPCRs (G protein-coupled receptors) in comparison to DDM [30], the utilization of LMNG has extended to numerous other classes of membrane proteins, as shown by our data and the existing literature [26]. A relevant practice worth highlighting is the addition of cholesteryl hemisuccinate (CHS) to detergent-solubilized MPs. CHS is a water-soluble cholesterol analog able to provide a more native and stabilizing environment for many purified MPs [32,33]. This addition of CHS has been reported mostly in combination with detergents from the MNG family, especially LMNG [74 of the 104 reports using MNG detergents (71%) contain CHS; Figure 3B].

Two other detergents that rank among the top four most commonly used surfactants for membrane protein extraction identified here are the steroidal detergents GDN and digitonin (6% and 5% of the reports, respectively). Digitonin is a natural steroidal saponin produced by the purple foxglove plant *Digitalis purpurea*. Its commercial form is, in fact, a mixture of about five different glycosides, with digitonin and digalonin, another saponin of similar structure, as the main components [34]. GDN is a recently developed synthetic variant of digitonin with some modifications. In digitonin, the head group comprises two galactoses, two glucoses, and a xylose, whereas GDN features a branched maltoside head group (Figure 4C and Figure 4D, respectively). Digitonin and GDN have CMC values of 0.25–0.50 mM and 0.018 mM, respectively. GDN, in particular, demonstrates higher homogeneity, enhanced water solubility, and lower toxicity compared to digitonin [26]. The presence of steroid hydrophobic groups in these detergents allows their usage in challenging cases involving membrane proteins that are unstable when solubilized with other classes of detergents [26]. In comparison to a previous report [22], we observed a decrease in digitonin usage (5% compared to 10% from the previous report) and an increase in the utilization of GDN (6% compared to 3% from the previous report).

### 3.2. Amphiphiles and Other Molecules Used in the Vitrification Step

**Figure 5:**
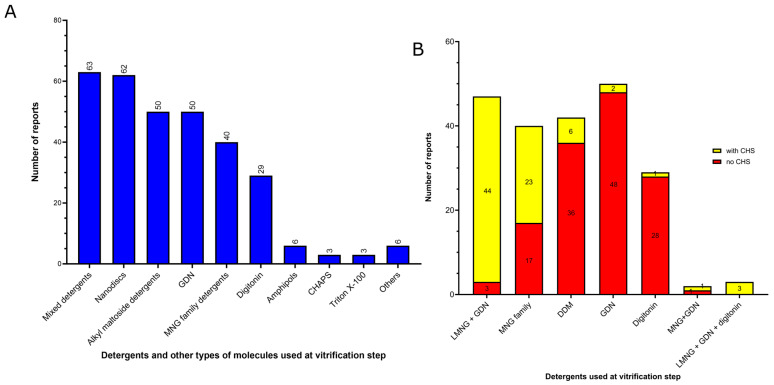
Profile of the surfactants and other types of molecules used in the vitrification step of membrane protein structures solved by using cryo-EM single-particle analysis below 3.0 Å resolution in the last two years (2021–2022) available in the Protein Data Bank. (**A**) Number of reports for each surfactant used in the vitrification step. Alkyl maltoside detergents include DDM and DM. MNG family detergents include LMNG and MNG. Mixed detergents include associations between different detergents. In this class, we notice the reports of the association between LMNG and GDN (48 reports), LMNG, GDN and digitonin (3 reports), MNG and GDN (2 reports), GDN and digitonin (2 reports), DM-NPG and digitonin (1 report), DDM and LMNG (1 report), DDM and Cymal-5 (1 report), DDM and Triton X-100 (1 report), DDM and CHAPS (1 report), LMNG and CHAPS (1 report), digitonin and sodium cholate (1 report), digitonin and LDAO (1 report). Nanodiscs include native nanodiscs (SMA copolymer nanodiscs, 5 reports), MSP-nanodiscs (50 reports), circularized nanodiscs (3 reports) and saposin–lipid–protein complexes (Salipro, 4 reports). Other surfactants include: LDAO (2 reports), Cymal-5 (1 report), C12E9 (1 report), glycerol (1 report), and peptidisc (1 report). (**B**) Analysis of the addition of cholesteryl hemisuccinate (CHS) on detergent-solubilized MPs at the vitrification step.

The six most classes of amphiphiles (or other types of molecules) present at the vitrification step of MP structures solved by using cryo-EM SPA at near-atomic resolution (better than 3.0 Å resolution) are shown in order of frequency of use (Figure 5A):

(1) Mixed detergents: 63 of 312 reports (20%). In this group, the most widely used association of detergents was the combination of LMNG and GDN, which is present in 47 of 63 reports.
(2) Nanodiscs: 62 of 312 reports (20%). In this group, the most widely used type is the membrane scaffold proteins (MSP)-based nanodiscs, which is present in 50 of 62 reports.
(3) Detergents with a single maltose-based polar moiety and single alkyl chain (DDM, UDM, DM): 50 of 312 reports (16%). In this group, the most widely used detergent is DDM, which is present in 42 of the 50 reports of this detergent class. DM is responsible for the eight remaining reports;
(4) Glyco-diosgenin (GDN): 50 of 312 reports (16%).
(5) Detergents belonging to the maltoside–neopentyl glycol (MNG) family (MNG and LMNG): 40 of 312 reports (13%). In this group, the most widely used detergent is LMNG, which is present in 38 of the 40 reports of this detergent class. MNG is responsible for the two remaining reports.
(6) Digitonin: 29 of 312 reports (9%).

These data indicate that a step of surfactant exchange or reconstitution into nanodiscs is often performed after extraction from the membrane and prior to vitrification of the membrane protein. While there is a clear predominance of DDM and LMNG usage during the protein membrane extraction step, no particular surfactant or other type of molecule exhibits distinct dominance during the vitrification step. There is a similar frequency of use between the mixed detergents, nanodiscs, DDM and GDN (20%, 20%, 16% and 16% of the reports, respectively). There is a significant increase in the usage of association between detergents in the vitrification step (20% of the reports) compared to the membrane protein extraction step (4.8% of reports, Figure 3A and Figure 5A, respectively). Notably, among the detergent associations used present in the vitrification step, the combination of LMNG and GDN stands out as the most prevalent, accounting for 47 out of the 63 reports (75%).

The combination of different detergents can be useful to explore their stabilizing properties of detergent–protein and detergent–detergent interactions and increase the effectiveness of the surfactant power obtained from this association without denaturing the membrane proteins. Strong detergent–protein interactions enhance membrane protein stabilization, and strong detergent–detergent interactions prevent protein aggregation and may minimize protein denaturation [26]. However, it is important to note that excessively strong detergent–protein interactions can lead to protein denaturation [35]. In the case of association between LMNG and GDN, LMNG interacts more strongly with the hydrophobic surface of MPs than DDM, GDN and digitonin due to its two alkyl chains, providing a strong detergent–protein interaction but without causing protein denaturation [26]. On the other hand, GDN has similar effects on protein–detergent interactions than DDM; however, the rigid hydrophobic group, resulting from the presence of a steroidal unit, promotes enhanced detergent–detergent interactions than LMNG [26]. It is important to note that the addition of CHS to detergent-solubilized MPs was mostly reported when the mixture of LMNG and GDN was employed at the vitrification step (44 out of the 47 reports, 93%) (Figure 5B). In comparison to the protein membrane extraction step, the addition of CHS is also extensively adopted in combination with detergents from the MNG family (23 out of 40 reports, 57%) (Figure 5B).

The selection of detergent and its concentration significantly impacts the structure, stability, and functionality of MPs. When detergent concentration is too low, MPs tend to aggregate and precipitate, whereas an excessive amount of detergent can lead to denaturation or dissociation of protein complexes [36]. In addressing these concerns, Size Exclusion Chromatography Multi-Angle Light Scattering (SEC-MALS) emerges as a highly suitable technique for assessing the molecular mass and the physicochemical heterogeneity of purified membrane protein–detergent complexes [37,38]. SEC-MALS integrates a SEC column that is connected in-line to detectors for ultraviolet (UV), light scattering (LS), and refractive index (RI) [37]. This comprehensive setup allows SEC-MALS to precisely determine the minimum amount of detergent required to maintain protein stability [38].

**Figure 6:**
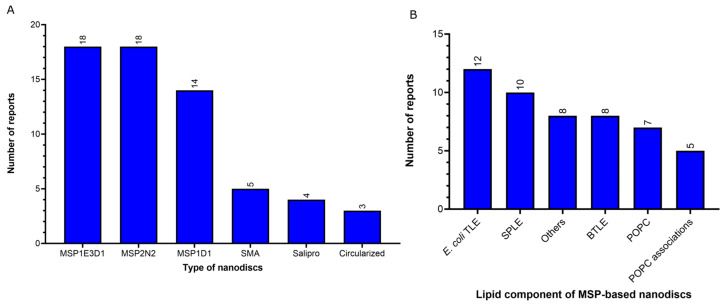
Profile of the type and composition of nanodiscs present on the vitrification step of membrane protein structures solved by using cryo-EM single-particle analysis below 3.0 Å resolution in the last two years (2021–2022) available in the Protein Data Bank. (**A**) Profile of the type of nanodiscs used in the vitrification step. Membrane scaffold proteins (MSP)-based nanodiscs: MSP1E3D1, MSP2N2, and MSP1D1. Salipro: saposin–lipid–protein complexes. SMA: Styrene–maleic acid copolymer (native nanodiscs). (**B**) Profile of the lipid component of MSP-based nanodiscs. *E. coli* TLE: Total lipid extract of *E.coli*. SPLE: soybean polar lipid extract. BTLE: brain total lipid extract. Others include POPG + POPE + cholesterol (1 report); BTLE + POPE (1 report); asolectin lipids (1 report), DOPC + DOPE + cardiolipin (1 report), yeast polar lipid extract (1 report); PC + PG + cholesterol (1 report), POPG (1 report), no info (1 report). POPC associations include: POPC + POPE + POPG (3 reports in 3:1:1 ratio), POPC + POPE + cholesterol (1 report), and POPC + POPG (1 report).

Nanodiscs offer detergent-free environments that represent another prominent type of preparation present in the vitrification step (20% of the reports) (Figure 5A), where the protein is reconstituted in a lipid environment [39]. While five reports of SMA copolymer nanodiscs (native nanodiscs) were identified at the protein membrane extraction step (Figure 3A), the vitrification step featured the same five reports of native nanodiscs in addition to 50 reports of membrane scaffold protein (MSP)-based nanodiscs, four of saposin–lipid-protein complexes (Salipro, Stockholm, Sweden) and three of circularized nanodiscs (Figure 5A and Figure 6A). MSP-based nanodiscs (50 of the 62 reports of nanodiscs, 80%) are small (7–50 nm in diameter) disc-shaped structures formed by a self-assembled bilayer of lipid bilayer (which mimic the native lipid bilayer) stabilized by two encircling amphipathic helical proteins derived from apolipoprotein A1 (ApoA1), called Membrane Scaffold Proteins (MSP) [39]. In contrast to other surfactants and molecules that are supplied by commercial sources, MSPs can be expressed in-house using *E.coli*. In addition, MSPs have been engineered to form nanodiscs with different transmembrane domain sizes, such as the MSPs identified here MSP1E3D1 (18 of the 50 reports of MSP-nanodiscs, 36%), MSP2N2 (18 of the 50 reports of MSP-nanodiscs, 36%) and MSP1D1 (14 of the 50 reports of MSP-nanodiscs, 28%) (Figure 6A). The first MSP, MSP1, was engineered with its sequence based on ApoA1, but without the globular N-terminal of its protein [40]. The MSP1D1 variant of MSP1 does not contain the first 11 residues of helix 1 and generates nanodiscs ~9.7 nm in diameter [41]. The MSP1E3D1 is a variant of MSP1 that contains an additional 3 helix sequences and generate nanodiscs of ~12.9 nm. MSP2N2 is a fusion of MSP2D1 and another MSP variant, MSP1D2, which features a complete deletion of the helix 1. MSP2N2 generates larger nanodiscs of 15.0–16.5 nm in diameter [41]. Regarding the lipid composition of the MSP-based nanodiscs of the MP structures solved cryo-EM SPA below 3.0 Å resolution, total lipid extracts of *E. coli* and soybean polar lipid extracts are the most widely used (12 and 10 of the 50 reports of MSP-nanodiscs, respectively) (Figure 6B). After the nanodiscs assembly, it is highly advisable to assess the quality of nanodiscs formation and determine the nanodiscs size. This can be accomplished through TEM of negative-stained samples, as incorrect lipid-to-MSP ratios during assembly can result in the formation of polydisperse particles resembling liposomes and larger aggregates when the ratio is too high or smaller lipid-scarce particles and free MSPs when the ratio is too small [42]. Determining the optimal lipid-to-MSP ratio is somewhat of a trial-and-error process. However, there are guidelines available to assist in determining the most suitable lipid-to-MSP ratio, taking into consideration factors such as the type of MSP used, the size of the protein, and the desired lipid composition for the nanodisc assembly [41,42,43].

Circularized nanodiscs (3 out of the 62 reports of nanodiscs) have been recently designed based on engineered scaffold proteins based on Apo1 scaffold protein where the N and C-terminus are covalently linked [44,45]. As a result of covalent circularization, larger (up to 80 nm diameter) and more homogeneous and stable nanodiscs were produced compared to the standard ones [45]. Two MP structures identified by this study were obtained at high resolution using circularizable scaffold protein NW9 (~8.5 nm of diameter) and one using NW11 (~11 nm of diameter). In terms of lipid composition, a report that used NW9 and a report that used NW11 employed soybean polar lipid extract. The NW9 remaining report utilized *E. coli* total extract lipid. Finally, 4 out of 61 reports of nanodiscs have been identified using a system related to MSP-nanodiscs, known as Salipro, wherein another protein, Saposin A, replaces MSPs. Regarding the lipid composition of MP structures that used Salipro at the vitrification step, two reports employed soybean polar lipid extract (one of them mixed with 25% cholesterol), while the other two reports used brain polar lipids.

Another strategy applied to create a realistic lipid environment for MPs while maintaining their physicochemical properties is to reconstitute them into vesicles or embed them in liposomes [28,46]. We did not identify any high-resolution report in the last two years (2021–2022) using these approaches. However, it is noteworthy that very recently (May 2023), the cryo-EM structure of a K^+^ channel embebed in plasma membrane vesicles at 2.7 Å resolution was presented [47].

Although nanodiscs have emerged as the predominant detergent-free environment of high-resolution MP structures, it is worth noting that two other detergent-free systems, namely amphipols and peptidiscs, have also been employed, albeit to a lesser extent. Similar to nanodiscs, amphipols and peptidiscs replace the detergent used for membrane protein extraction prior to the sample vitrification. Among the 312 unique reports analyzed, amphipols were employed in the vitrification step in six cases (Figure 5A). Amphipols represent a class of short and flexible amphipathic polymers that directly bind to the hydrophobic surface of MPs and effectively replace detergents [48,49]. Unlike detergents that require 50–200 to associate with a single MP, only a small number of amphipol molecules are needed to cover the entire hydrophobic surface of a MP [26,50]. Moreover, amphipols can be used to stabilize protein complexes and facilitate the analyses of fragile MPs and MP complexes [48,51]. Amphipols can also be useful to produce MPs, most noticeably by assisting their folding from the denatured state obtained after solubilizing MP inclusion bodies in either SDS or urea [48,51]. Between the six reports identified here as having amphipol usage in the vitrification step, five used A8-35 and one used PMAL-C8, although other types of amphipols can also be used for MP structural studies [48]. Even when examining the vitrification step of MP structures resolved at lower resolution ranges (3–5 Å) in the past two years, the usage of amphipols remained limited, with a low number of reports (3), which may be due to their cost. However, amphipols have the potential to be further explored by the MP structural biology community. Amphipols manipulation and transitioning from detergents to amphipols are simpler processes compared to the preparation of nanodiscs, which requires the expression and purification of the MSP and a correct assemblage of lipids, MP and MSP while maintaining an optimal lipid-to-MSP ratio. Moreover, because a small quantity of amphipols can efficiently cover the entire hydrophobic surface of a MP, they do not form a heavy layer of detergent or lipid nanodiscs around the transmembrane domain of MPs. This belt can introduce some difficulties in terms of the analysis of the transmembrane domain during the cryo-EM data processing workflow, requiring the use of masks of these belts to refine the final cryo-EM maps. The utilization of amphipols eliminates the need for these masks, thereby streamlining cryo-EM data processing.

In addition, a single report was found regarding the use of peptidiscs (classified within “others” in Figure 5A) among the MP structures resolved at below 3.0 Å resolution. Peptidiscs constitute a detergent-free surfactant system very recently developed (2018) that uses multiple copies of a short sequence of a unique helical peptide (NSP_r_) redesigned to have optimal hydrophobic and hydrophilic properties. These helical peptides wrap around the hydrophobic transmembrane domains of the MPs and shield them from the watery solution [52].

**Figure 7:**
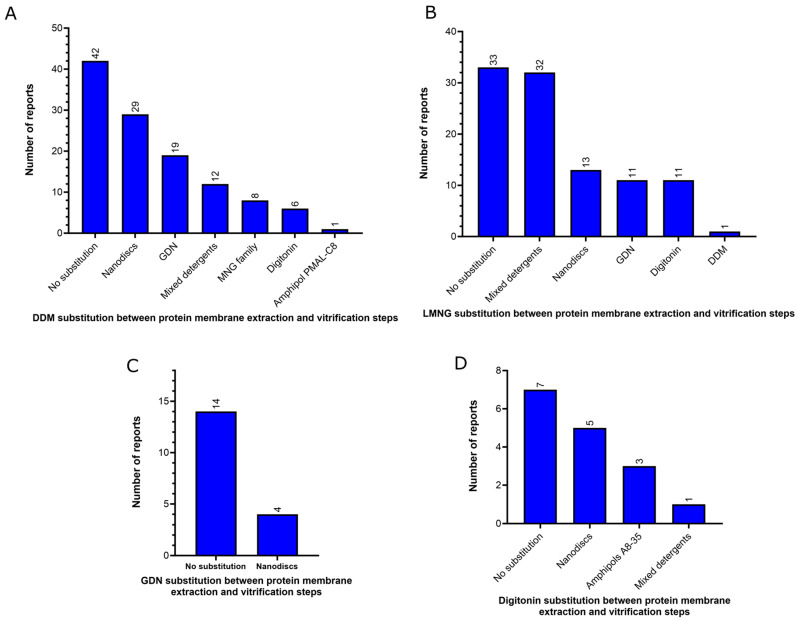
Profile of detergent substitution between protein membrane extraction and vitrification steps of membrane protein structures solved by using cryo-EM single-particle analysis below 3.0 Å resolution in the last two years (2021–2022) available in the Protein Data Bank. Numbers of DDM (**A**), LMNG (**B**), GDN (**C**) and digitonin (**D**) substitution between protein membrane extraction and vitrification steps. Mixed detergent reports in panel A include: LMNG and GDN (10 reports), MNG and GDN (1 report), DDM and Cymal-5 (1 report). Mixed detergent reports in panel B include: LMNG and GDN (30 reports) and LMNG, GDN and digitonin (2 reports). Mixed detergent reports in panel D include 1 report of GDN and digitonin.

As mentioned above, a surfactant exchange or nanodiscs reconstitution step is frequently realized after membrane extraction and prior to vitrification of the membrane protein samples. There is a strong decrease in the use of DDM and LMNG (when used alone, without mixing with another detergent) in the vitrification step. Analysis of the substitutions among the four most common surfactants (DDM, LMNG, GDN and digitonin) used for membrane protein extraction revealed that DDM had the highest rate of exchange prior to the vitrification steps. Out of the 117 unique reports that used DDM for membrane protein extraction, only 42 retained it for the vitrification step (Figure 7A), representing a 65% replacement of DDM. The most frequently used substitute for DDM in the vitrification step was nanodiscs (29 unique reports), followed by GDN (19 unique reports), association between detergents (12 unique reports), detergents from the MNG family (8 unique reports), digitonin (6 unique reports), and amphipol PMAL-C8 (one unique report) (Figure 7A). Interestingly, in all cases but one, where DDM was present in the vitrification step, it was also employed for protein extraction from the membrane. Regarding LMNG, of the 101 unique reports that used this detergent for membrane protein extraction, 33 retained it as the sole surfactant in the vitrification step (Figure 7B). Although the use of LMNG alone has significantly decreased in the vitrification step, it has not been completely replaced. Instead, in 32 unique reports, LMNG was used in combination with other detergents, with 30 reports including a combination of LMNG and GDN and 2 combining LMNG with both GDN and digitonin. Thus, LMNG was completely exchanged before sample vitrification in 36 out of the 101 unique reports (35% of total replacement). The most common substitute for LMNG in the vitrification step was nanodiscs (13 unique reports), followed by GDN (11 unique reports), digitonin (11 unique reports) and DDM (1 unique report) (Figure 7B). GDN was less exchanged between membrane protein extraction and the vitrification steps. Out of the 18 unique reports that utilized this detergent for membrane protein extraction, it was exchanged before sample vitrification in only four cases, all of which were nanodiscs (22% replacement) (Figure 7C). Regarding digitonin, out of the 16 unique reports that used this detergent for membrane protein extraction, 7 retained it as the sole surfactant in the vitrification step, and 1 used it in combination with GDN. In three cases, digitonin was replaced by Amphipols A8-35, and in five cases, it was replaced by nanodiscs before sample vitrification (50% of total replacement) (Figure 7D). This high level of detergent exchange for detergent-free environments, particularly MSP-based nanodiscs, can be attributed to the complications that detergents can cause when aiming for high-quality cryo-EM images, including the introduction of detergent artifacts, increased vitrified ice thickness, and particle distribution and preferred orientational issues [53].

The attachment of small Fab fragments [54], nanobodies [55], or, more recently, megabodies [56] to small proteins is a strategy used to overcome size-related limitations in the application of cryo-EM SPA for smaller proteins. Small proteins generally suffer from low SNR, primarily because SNR is influenced by the vitreous ice thickness relative to the particle size. Additionally, small proteins intrinsically lack distinctive morphological features, which impairs the particle alignment needed for protein reconstructions. Furthermore, some of these small Fab fragments and nanobodies play a pivotal role in stabilizing protein complexes, especially GPCR-G protein complexes. Indeed, in this study, we observed a high level of usage of these molecules for the obtention of GPCR structures. We identified that 65 out of 75 GPCR high-resolution structures were resolved through binding to antibodies or nanobodies. Specifically, 35 GPCRs reports (46%) were resolved in complex with scFV16 antibody fragment, 21 (28%) with nanobody 35 (Nb35), 8 (11%) with both Nb35 and scFV16, 1 (1.3%) with Nb35 and M22Fab, 1 (1.3%) with nanobody 6, and 1 (1.3%) with scFV16 and other Fab fragments. We also identified 10 reports of using these binding partners to stabilize protein complexes or increase the particle size in other MP families outside the GPCR category. Specifically, we found four reports of nanobodies (NbC1, Nb26, Nb872 + Nb881, and NbMsbA) bound to the structure of a channel, a MFS transporter, a cellulose synthase and an ABC transporter, respectively. We also identified three reports of megabodies (Mb25, Mb177 and c7HopQ bound to the structure of a Cys-loop receptor, SLC and *O*-acetyltransferase, respectively. Additionally, we identified three reports of Fab fragments bound to two structures of *O*-acetyltransferase and one structure of a MFS transporter. Interestingly, among the seven reported MP structures with a total structure weight smaller than 100 kDa where none of those additional binding partners, four of them used nanodiscs (three MSP-based nanodiscs and one circularized NW9 nanodisc) in the vitrification step. This suggests that nanodiscs may also be a useful tool to increase the particle size, rendering small MPs suitable for cryo-EM SPA.

The usage profile of detergent, nanodiscs and other molecules in the vitrification step presented in this study focused on the analysis of high-resolution cryo-EM structures obtained between 2021 and 2022 is quite different from a previous report conducted two years ago [22], which analyzed cryo-EM structures solved at all resolution ranges. In this previous report, digitonin was the most preferred choice at the vitrification step, accounting for 18% of the reports [22]. However, in the current study, digitonin is only the sixth most frequent choice at the vitrification step (Figure 5A) and accounts for 9% of the reports. On the other hand, we observed a substantial increase in the usage of mixed detergents, particularly those containing LMNG, in comparison to the previous report. We identified in the present study that mixed detergents were the preferred choice for sample vitrification, comprising 20% of the reports (Figure 5A), whereas in the previous study, mixed detergents accounted for only 5% of the reports [22]. Furthermore, we noted a slight increase in the usage of GDN in the current report in comparison to the previous one, with this detergent representing 16% of the reports, as opposed to 11% in the previous study. The usage of single-maltose detergents (DDM, UDM, DM), detergents from the MNG family and MSP-based nanodiscs were similar in both studies (~17%, ~13% and ~16% of the reports, respectively). Regarding the exchange of the amphiphiles between membrane protein extraction and vitrification steps, both studies observed a similar replacement rate of DDM by other detergents or nanodiscs (65–68%), and digitonin was found to be the least exchanged detergent. However, a notable difference was observed between the two studies concerning LMNG. Whereas a previous study observed a high level of LMNG substitution for other detergents or amphiphiles before sample vitrification (~59% of the reports) [22], here we observed that LMNG was maintained in the vitrification step, alone or in combination with other detergents, particularly GDN (Figure 7B).

A frequently employed strategy to improve the quality of the samples for cryo-EM SPA is the addition of some specific detergents before the vitrification step to overcome common problems observed in the vitrified samples, such as protein aggregation, high particle adsorption at the air–water interface, and, preferential particle orientation [7,11,53]. Through the cryo-EM sample preparation, after the sample is deposited onto the grids prior to freezing, a significant number of protein molecules diffuse to the air–water interface within milliseconds. This phenomenon can lead to certain protein regions preferentially binding to the air–water interface, resulting in protein particles molecules aligning in a preferred orientation [57,58]. This orientation bias hinders the achievement of high-resolution structures and adversely affects the reliability and interpretability of the obtained structures [58,59]. Also, protein molecules adsorbed onto the air–water interface may suffer partial damage or even form a layer of denatured protein [57,58]. Here, we were able to identify five reports where fluorinated detergents (three using fluorinated fos-choline−8 at 1.5–3 mM, and two using fluorinated octyl maltoside, FOM, at 60 μM and 5 mM, respectively) and one where a high CMC detergent (0.2% CHAPS) were added before vitrification step to mitigate these issues. Finally, we also identified one report that showed that megabodies can prevent preferential particle orientation [56].

## 4. Sample Vitrification

The vitrification step consists of applying approximately 3 μL of protein solution onto a grid, followed by blotting with filter paper to eliminate excess solution and create a very thin layer of protein suspension on the grid. The grid is then rapidly frozen by plunging it into liquid ethane cooled by liquid nitrogen [6]. Plunge freezing could be performed manually or using some commercial devices, such as Vitrobot^TM^ (Thermo Fischer Scientific^TM^, Waltham, MA, USA), EM GP2 (Leica Microsystems GmnH, Wetzlar, Germany) and Cryoplunge^TM^ 3 (Gatan, Inc., Pleasanton, CA, USA). In our analysis, we identified 285 reports that used Vitrobot^TM^ (94%), 16 that used EM GP2 (5.2%), 1 report that used Cryoplunge^TM^ 3 (0.3%), and 1 report that performed the blotting and plunge freezing manually (0.3%).

However, optimal ice thickness is difficult to reproduce from one grid to another due to the uneven surface properties of the blotting papers [60]. Moreover, the dwell time of the samples on the grids before freezing at the level of seconds is sufficient to favor particle adsorption at the air–water interface [61]. In order to solve these limitations, several efforts have been made recently to develop alternative blotting-free methods that guarantee a more reliable and reproducible grid preparation and that diminish the deleterious effects of the air–water interface. For these blotting-free methods, several automated devices have been developed employing inkjet printing (Spotiton) [62], gas pressure spray (microfluidic devices) [63,64], ultrasonic spray [65,66,67], electrostatic spray [68], and sample scribing [69,70] (for a detailed description of these methods, see [11]). Here, we did not identify the use of any of these new blotting-free methods for the obtention of MP cryo-EM structures at high resolution, likely due to the limited commercial availability of most of these new devices. However, very recently, the commercialized form of Spotiton, known as Chamaleon (SPT Labtech Ltd., Covina, CA, USA) [71], has been acquired by several cryo-EM facilities worldwide. This system employs an inkjet mechanism to spray picoliter-sized droplets of the sample solution are sprayed onto self-wicking nanowire grids. The entire sample volume can be accurately dispensed through the piezoelectric inkjet head by applying voltage pulses to control ice thickness [62,72,73]. This method potentially provides more reproducible grid preparations than blotting-dependent methods and reduces the dwell time on the grids before freezing for seconds to hundreds of milliseconds [74]. This minimizes the number of particles adsorbed at the air–water interface and increases the number of particles adsorbed in the vitreous ice. Consequently, particle distribution is enhanced in the grids, and the preferred particle orientation problem is alleviated [72,74]. The Chamaleon system has been tested in several cryo-EM facilities. For example, vitrification experiments using Chamaleon conducted with soluble proteins demonstrated improved particle distribution onto the grids and enhanced the stability and reduced dissociation level of a protein/DNA complex compared to gold grids [75]. In the future, it will be possible to evaluate its overall impact in the cryo-EM field in terms of quality gains in sample vitrification and in the collected micrographs from several types of samples, including MPs.

## 5. Type of the Grids

Grids serve as the sample carriers for TEM and cryo-EM. They have a standard diameter of 3 nm and typically consist of two main components: a mesh base and a foil [11]. The mesh base is made of a metal that is capable of offering mechanical stability, electron beam conduction and heat dissipation. The foil coats the grid by being placed on top of this mesh and contains micrometer-sized holes that support the vitreous ice with the embedded protein particles and allow electron passage during microscope imaging [76]. The foil can contain a regular repeating array of circular holes (Quantifoil^®^ or C-flat^TM^ grids, Großlöbichau, Germany) or an irregular geometry of holes (Lacey grids) [6,11]. These varying designs of mesh base and foils meet specific experimental needs and preferences in TEM and cryo-EM applications.

**Figure 8:**
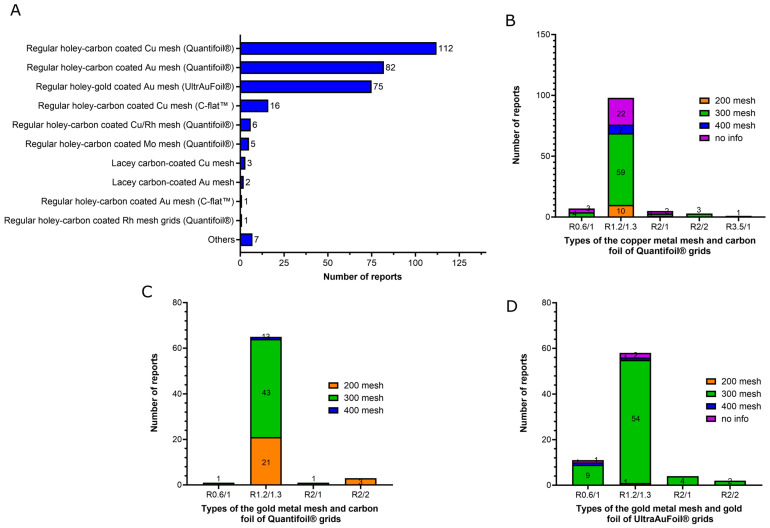
Profile of the grids used for determination of membrane protein structures solved by using cryo-EM single-particle analysis below 3.0 Å resolution in the last two years (2021–2022) available in the Protein Data Bank. (**A**) Type of the grids employed; (**B**) types of the cooper metal mesh and carbon foil of Quantifoil^®^ grids; (**C**) types of the gold metal mesh and carbon foil of Quantifoil^®^ grids; (**D**) types of the gold metal mesh and gold foil of UltrAuFoil^®^ grids.

The three major types of the grids used for cryo-EM data collection of MP solved by using cryo-EM SPA at near-atomic resolution (below 3.0 Å resolution) in the last two years (2021–2022) are in order of frequency of use (Figure 8A):

(1) Regular holey-carbon coated copper mesh grids (Quantifoil^®^): 112 of 310 reports (36%);
(2) Regular holey-carbon coated gold mesh grids (Quantifoil^®^): 82 of 310 reports (26.5%);
(3) Regular holey-gold coated gold mesh grids (UltrAuFoil^®^): 75 of 310 reports (24%).

Copper mesh grids are the most popular choice identified in this study, as would be expected as they are the historically most popular grids used in cryo-EM [76]. Copper mesh grids are inexpensive and display good electron conductivity, which is an important feature in preventing detrimental sample charging of the samples, which would lead to a significant loss in terms of micrograph quality [6,7,11]. Gold mesh grids are even more conductive [15], although they are more expensive. Concerning the foil, amorphous carbon-film-coated grids are also the most popular choice for cryo-EM SPA of MP identified here, as well as for cryo-EM in general, because they are also inexpensive and easily manufactured into foils [6,11,76]. However, they have challenging limitations [77,78]. The difference in thermal contraction between the carbon foil and the metal mesh (copper, gold and other metals) at cooled temperatures during vitrification may shrink the carbon foil and induce deformations in the ice layer. This results in significant beam-induced motion during microscope imaging, resulting in a loss of resolution in the micrographs [77,78]. The substitution of carbon films for gold films on gold mesh grids (UltrAuFoil^®^ grids) results in an entire grid structure composed of the same material that eliminates the retraction of the foil caused by the differential thermal contraction at cooled temperatures. As a result, a drastic reduction in beam-induced motion during microscope imaging is achieved, significantly improving micrograph quality [77,78]. Besides the 75 UltrAuFoil grid reports identified here, we noted two reports that used homemade nanofabricated holey-gold grids with holes of 1.5 and 2 μm, respectively, and two reports that employed CryoMatrix^®^ gold grids. Recently, it was demonstrated that an amorphous nickel–titanium alloy film has similar effects in reducing beam-induced motion and improving image quality than gold films. Here, we identified two reports that employed this film (CryoMatrix^®^ nickel–titanium alloy film).

C-flat^TM^ grids have demonstrated good performance in the cryo-EM field as they are potentially able to provide, thanks to their ultra-flat surface architecture, even lower ice thickness and better particle dispersion in grids compared to Quantifoil^®^ grids [79,80]. Despite these advantages, they did not represent a popular choice in terms of cryo-EM of MP at near-atomic resolution, as evidenced by their usage in only 17 of 320 reports (5.3%) identified here (16 reports of C-flat^TM^ holey-carbon coated copper mesh grids and 1 report of homemade C-flat^TM^ holey-gold coated gold mesh grids) (Figure 8A). Grids with irregular geometry of the holes (Lacey grids) were only used in six reports (1.8%), five being for carbon-coated grids and one report for gold-coated grids (Figure 8A), although these grids are quite popular for the cryo-EM analysis in general.

Foil films can be categorized based on the diameter of the holes and the width between them, while metal meshes are classified by the number of squares per inch. For instance, the notation R1.2/1.3 denotes a foil film with a hole size of 1.2 μm and a distance of 1.3 μm between the holes. The advantage of using foils with larger holes is the possibility of collecting more images per hole, resulting in faster automated data collection. However, larger hole sizes increase the chances of obtaining deformation and inhomogeneity in the ice layer, resulting in beam-induced motion of the specimen and producing blurred images [15]. The use of smaller distances between the holes reduces the amount of foil on the metal support of the grids, which enhances the chances of the protein particles entering the holes. However, it results in more fragile grids that require more careful sample preparation manipulation. Among the three major types of grids used for cryo-EM SPA of MP proteins identified in this study, the most typical foil films chosen were R1.2/1.3 (77–94% of the reports). All other reports used R2/1, R2/2 and R0.6/1 foils, except for a single case, where R3.5/1 foils were used (Figure 8B–D). The choice of using 0.6–2 μm hole size foils can be attributed to the nature of the samples deposited on the grids, which did not require larger foil holes. Regarding the metal mesh, a grid designed as 400 mesh indicates that it contains 400 squares per inch. Smaller mesh grids consequently have larger squares with a greater number of foil holes, which makes the setting of the automated data collection faster and easier. For example, a 100 mesh grid contains squares of 204 μm and 6659 foil holes per grid square, while a 400 mesh grid contains squares of 38 μm and 231 foil holes per grid square [81]. On the other hand, the smaller the mesh, the more fragile the grid, which requires very careful and delicate handling of the grid during sample preparation. In this study, we noticed that the cryo-EM community of MPs has a preference for Quantifoil^®^ and UltrAuFoil^®^ grids of 300 mesh (Figure 8B–D).

A common challenge in cryo-EM sample preparation is the propensity of certain samples to adsorb onto the carbon film rather than entering the foil holes. This behavior results in the collection of an insufficient number of particles to achieve high-resolution structures [6,7,11]. To address this issue, a commonly used strategy involves creating conditions that promote the entry of protein particles into the holes by adding a continuous film on top of the foil. This provides an extra physical support for the protein particles to adsorb onto [6,7,11]. Moreover, these additional films can also improve particle distribution on the grid and significantly decrease the preferential particle orientation issue. Here, we identified right reports where a thin layer of continuous carbon film (2–4 nm) was added on top of the grids, five being on carbon-coated copper mesh grids (Quantifoil^®^), two on carbon-coated gold mesh grids (Quantifoil^®^) and one on UltrAuFoil^®^ grids. We also identified eleven reports where the grids were covered with a layer of graphene oxide, five being on carbon-coated copper mesh grids (Quantifoil^®^), five on carbon-coated gold mesh grids (Quantifoil^®^) and one on Lacey carbon-supported copper grids. The thin carbon layer reduces the signal-to-noise ratio of micrographs, leading to the loss of high-resolution information, particularly for small proteins. On the other hand, this problem is not observed with the introduction of the graphene oxide layer [82,83]. Finally, we identified one report of a lysine-coated graphene oxide layer applied to carbon-coated copper mesh grids (Quantifoil^®^). This extra layer of lysine is very effective in attracting negatively charged particles onto the grid surface [84].

## 6. Conclusions and Perspectives

The present study offers an overview of the prevailing choices for the most critical parameters in sample preparation for the obtention of MP structures at near-atomic resolution through the use of cryo-EM SPA over the past two years. Consequently, we can share our current perspective on future developments in the field with the MP structural biological community.

We observed that DDM continues to be the most commonly used surfactant for the extraction of MPs from the membrane. However, its prominent dominance appears to have decreased in recent years, as we noticed a significant increase in the use of LMNG for membrane protein extraction, often combined with the cholesterol analog, CHS. We also observed that there is a high rate of substitutions between the types of surfactants used for membrane protein extraction and those present in the sample vitrification step. These detergents are being replaced in the vitrification step mainly by detergent-free environment MSP-based nanodiscs and by a mixture of detergents containing LMNG, GDN and the cholesterol analog CHS. On the other hand, the potential development of new detergents that focus on enhancing detergent–detergent interactions by promoting hydrogen-bonding interactions between detergent molecules [26], which can provide new surfactants with higher membrane extraction efficiency and enhanced protein-stabilizing effects. Additionally, the recent discovery of two small diglucoside amphiphiles able to form discoidal assemblies containing lipids similar to the nanodiscs [85] could impact the field in the future. This discovery may open possibilities for obtaining new surfactants, where any surfactant exchange would be unnecessary between membrane protein extraction and vitrification steps. However, other detergent-free environments, such as amphipols, circularized nanodiscs, and peptidiscs, have produced excellent results for high-resolution membrane protein structure determination through the use of cryo-EM SPA. Nevertheless, as evidenced in this study, their use remains little widespread. Amphipols are easier to manipulate than other detergent-free environments and did not form a detergent or lipid belt around the transmembrane domain that requires masking of these belts along the cryo-EM data processing. Moreover, samples in amphipols produced more uniform ice films on the grids and, therefore, a better particle distribution in comparison to detergents [86]. Recently, a new software called EMReady v1.0 has become available [87], offering the potential to significantly enhance the interpretability of the cryo-EM maps. EMReady has shown promising results in effectively suppressing the lipid nanodisc layer in a specific cryo-EM data set. In this particular data set, the visibility and analysis of the transmembrane domain of a particular MP had been entirely obstructed by this layer, and EMReady successfully rendered this layer almost invisible after processing the cryo-EM map. As a result, this innovative software holds great promise for improving the cryo-EM data processing workflow of MPs by mitigating the noise introduced by heavy detergent or lipid nanodisc layers. The MP structural biology community should explore the capabilities of this software on a larger scale and evaluate its effectiveness across different cryo-EM datasets.

All reports analyzed here employed the blotting technique for sample vitrification, with a clear predominance for utilizing the Vitrobot^TM^ automated device. Moreover, analysis of the reports showed a dominant trend in using cooper or gold 300 mesh grids coated with regular holey-carbon (Quantifoil^®^) or holey-gold (UltrAuFoil^®^) R1.3/1.2 foils. Despite the good performance of the C-flat^TM^ grids in obtaining high-resolution cryo-EM structures, their utilization is not yet widespread in the MP field. The popularization of the recent new blotting-free methods for sample vitrification, such as Chamaleon and others, holds the potential to make obtaining high-resolution structures more routine in the future. The initial results pertaining to the application of blotting-free methods in the vitrification of some classes of soluble proteins have demonstrated a reduction in particle adsorption in the air–water interface, the mitigation of the preferred orientation problem, and a reduction in the dissociation level of protein complexes [71,75]. These outcomes should encourage the MP structural biological community to consider scaling up the utilization of these methods. This will allow the replication of these results in the vitrification of MPs and the systematic evaluation of the unique features and limitations of the blotting-free methods in the vitrification of MPs in various amphiphilic environments or membrane mimetics.

Other strategies to decrease the particle adsorption in the air–water interface and mitigate the preferential orientation problem include the addition of some specific detergents to the samples before vitrification. These detergents form a protective layer at the air–water interface, effectively preventing protein adsorption and denaturation there [53]. Here, we identified a few cases where the addition of fluorinated detergents and high CMC detergents proved effective in addressing the air–water interface challenges. The use of this strategy could be further explored by the MP cryo-EM community, especially in samples without any detergent (nanodiscs, amphipols) or with detergents with very low CMC values (LMNG). Strategies to prevent the particle from sticking to the foil films that can also mitigate the preferential orientational problem were also identified in this study. We identified some cases where a thin layer of continuous carbon film or graphene oxide was applied to avoid the preferred orientation. However, other successful strategies aimed at the same purpose in the cryo-EM studies of different proteins, such as cryo-EM grids with Ni-NTA functionalized films to bind histidine-tagged proteins [88,89], 2D streptavidin crystal films [90] or PEGylation treatment of the grids [91] or of the protein samples [92], were not identified in this study. These strategies may have limited awareness within the MP cryo-EM community and could be further explored in the future.

Our analysis also demonstrated that the attachment of binding partners, such as Fab fragments, nanobodies or megabodies, has become a common strategy to address the challenges posed by small proteins when applying cryo-EM SPA. We identified that this strategy was employed not only in GPCRs but also in a significant number of reports across various MP families. We also observed that nanodiscs could be a valuable approach to increasing the protein particle size, particularly with the recent introduction of circularized nanodiscs that can generate larger nanodisc assemblies compared to MPS-based nanodiscs [45]. These approaches highlight the potential of cryo-EM SPA application to determine high-resolution structures of small MPs.

An exciting feature of cryo-EM SPA is its capacity to capture images representing various conformational states of the same macromolecule because of the rapid freezing of protein in solution [93]. Consequently, cryo-EM SPA can provide a window into the dynamics properties of proteins, revealing different conformational states and enabling inferences about how a structure transitions from one conformation to another. In this context, a notable trend in the cryo-EM SPA field in recent years is the development of several different pieces of software to extract protein dynamics and continuous conformational flexibility data from the cryo-EM image datasets, such as 3DVA [94], MSPACE [95], e2gmm [96], Scipion-EM-ProDy workflow [97] and others. Additionally, recently released software advancements have enhanced the flexible fitting of atomic structural models into cryo-EM maps through molecular dynamic (MD) simulations, such as Namdinator [98] or by approaches that combine normal mode analysis and MD simulations, such as NMMD [99] and MDeNM-EMfit [100]. These latter approaches are particularly noteworthy as they are able to perform a comprehensive exploration of the free energy landscapes of large conformational changes. Consequently, it becomes possible to resolve various states of a given structure in atomic detail. Therefore, the pursuit of high-resolution MP structures serves not only to provide intricate structural details but also to yield profound insights into their functional dynamics and underlying mechanisms.

Despite the remarkable advances in cryo-EM SPA in recent years, it remains an evolving technique. The continuous development of novel sample preparation approaches has the potential to usher in groundbreaking progress in the structural knowledge at the near-atomic level of an increasing number of MPs. Moreover, it can offer novel, accessible experimental conditions for obtaining high-resolution structures of challenging samples and large membrane protein complexes.

## Data Availability

Not applicable.

## Supplementary Materials

The following supporting information can be downloaded at: https://www.mdpi.com/article/10.3390/ijms241914785/s1.

## Abbreviations

ABC	ATP-binding cassette
APC	Amino Acid/Polyamine/Organocation
ApoA1	Apolipoprotein A1
C12E9	Nonaethylene glycol monododecyl ether
C12E8	Octaethylene glycol monododecyl ether
CCD	Charged-coupled device
CHAPS	3-[(3-cholamidopropyl)dimethylammonio]-1-propanesulfonate
CHS	Cholesteryl hemisuccinate
CMC	Critical micelle concentration
CMOS	Complementary metal–oxide–semiconductor
Cryo-EM	Cryo-electron microscopy
Cymal-4	4-cyclohexyl-1-butyl-β-D-maltoside
Cymal-6	6-cyclohexyl-1-hexyl-β-D-maltoside
DDD	Direct detector device
DDM	n-Dodecyl-β-D-maltopyranoside
DM	Decyl-b-D-Maltopyranoside
DQE	Detective quantum efficiency
FOM	Fluorinated octyl maltoside
FAB	Fragment antigen binding
GDN	Glyco-diosgenin
GPCR	G-protein coupled receptor
LDAO	N,N-Dimethyl-n-dodecylamine N-oxide
LMNG	Lauryl Maltose Neopentyl Glycol
MFS	Major facilitator superfamily
MNG	Maltose-Neopentyl Glycol
MP	Membrane Protein
MSP	Membrane Scaffold Protein
OGNG	Octyl glucose neopentyl glycol
t-PCCαM	4-trans-(4-trans-Propylcyclohexyl)-cyclohexyl α-maltoside
PDB	Protein Data Bank
POPC	1-palmitoyl-2-oleoyl-glycero-3-phosphocholine (16:0–18:1PC)
POPE	1-palmitoyl-2-oleoyl-sn-glycero-3-phosphoethanolamine (16:0–18:1PE)
POPG	1-palmitoyl-2-oleoyl-sn-glycero-3-phosphoglycerol (16:0–18:1PG)
PTS	Phosphoenolpyruvate-dependent phosphotransferase
Salipro	Saposin–lipid–protein complexes
SLC	Solute carrier
SMA	Styrene maleic acid copolymer
SPA	Single-particle analysis
SPME	Soybean polar lipid extract
SNR	Signal-to-noise ratio
SSS	Solute sodium symporter
TEM:	Transmission electron microscopy
TLE:	Total lipid extract
UDM	n-Undecyl-β-D-maltopyranoside
