# Supplementary material for: Overview of Membrane Protein Sample Preparation for Single-Particle Cryo-Electron Microscopy Analysis
Source: Int J Mol Sci. 2023 Sep 30;24(19):14785. doi: 10.3390/ijms241914785 (PMC10573263; doi:10.3390/ijms241914785)
Supplement: Supplementary file 1 [file ijms-24-14785-s001.zip › ijms-2581590-supplementary.pdf]

Supplementary Material
List of all manuscripts evaluated

1. Klusch, N.; Senkler, J.; Yildiz, Ö.; Kühlbrandt, W.; Braun, H.-P. A Ferredoxin Bridge Connects the Two Arms of Plant Mitochondrial Complex I. *The Plant Cell* **2021**, *33*, 2072–2091, doi:10.1093/plcell/koab092.
2. Schimpf, J.; Oppermann, S.; Gerasimova, T.; Santos Seica, A.F.; Hellwig, P.; Grishkovskaya, I.; Wohlwend, D.; Haselbach, D.; Friedrich, T. Structure of the Peripheral Arm of a Minimalistic Respiratory Complex I. *Structure* **2022**, *30*, 80-94.e4, doi:10.1016/j.str.2021.09.005.
3. Yan, R.; Zhang, Y.; Li, Y.; Ye, F.; Guo, Y.; Xia, L.; Zhong, X.; Chi, X.; Zhou, Q. Structural Basis for the Different States of the Spike Protein of SARS-CoV-2 in Complex with ACE2. *Cell Res* **2021**, *31*, 717–719, doi:10.1038/s41422-021-00490-0.
4. Schureck, M.A.; Darling, J.E.; Merk, A.; Shao, J.; Daggupati, G.; Srinivasan, P.; Olinares, P.D.B.; Rout, M.P.; Chait, B.T.; Wollenberg, K.; et al. Malaria Parasites Use a Soluble RhopH Complex for Erythrocyte Invasion and an Integral Form for Nutrient Uptake. *eLife* **2021**, *10*, e65282, doi:10.7554/eLife.65282.
5. Montgomery, M.G.; Petri, J.; Spikes, T.E.; Walker, J.E. Structure of the ATP Synthase from *Mycobacterium Smegmatis* Provides Targets for Treating Tuberculosis. *Proc. Natl. Acad. Sci. U.S.A.* **2021**, *118*, e2111899118, doi:10.1073/pnas.2111899118.
6. Kolata, P.; Efremov, R.G. Structure of Escherichia Coli Respiratory Complex I Reconstituted into Lipid Nanodiscs Reveals an Uncoupled Conformation. *eLife* **2021**, *10*, e68710, doi:10.7554/eLife.68710.
7. Conners, R.; McLaren, M.; Łapińska, U.; Sanders, K.; Stone, M.R.L.; Blaskovich, M.A.T.; Pagliara, S.; Daum, B.; Rakonjac, J.; Gold, V.A.M. CryoEM Structure of the Outer Membrane Secretin Channel pIV from the F1 Filamentous Bacteriophage. *Nat Commun* **2021**, *12*, 6316, doi:10.1038/s41467-021-26610-3.
8. Budiardjo, S.J.; Stevens, J.J.; Calkins, A.L.; Ikujuni, A.P.; Wimalasena, V.K.; Firlar, E.; Case, D.A.; Biteen, J.S.; Kaelber, J.T.; Slusky, J.S. Colicin E1 Opens Its Hinge to Plug TolC. *eLife* **2022**, *11*, e73297, doi:10.7554/eLife.73297.
9. Ye, F.; Xu, L.; Li, X.; Zeng, W.; Gan, N.; Zhao, C.; Yang, W.; Jiang, Y.; Guo, J. Voltage-Gating and Cytosolic Ca^{2+} Activation Mechanisms of *Arabidopsis* Two-Pore Channel AtTPC1. *Proc. Natl. Acad. Sci. U.S.A.* **2021**, *118*, e2113946118, doi:10.1073/pnas.2113946118.
10. Su, C.-C.; Lyu, M.; Morgan, C.E.; Bolla, J.R.; Robinson, C.V.; Yu, E.W. A 'Build and Retrieve' Methodology to Simultaneously Solve Cryo-EM Structures of Membrane Proteins. *Nat Methods* **2021**, *18*, 69–75, doi:10.1038/s41592-020-01021-2.
11. Thom, C.; Ehrenmann, J.; Vacca, S.; Waltenspühl, Y.; Schöppe, J.; Medalia, O.; Plückthun, A. Structures of Neurokinin 1 Receptor in Complex with G_q and G_s Proteins Reveal Substance P Binding Mode and Unique Activation Features. *Sci. Adv.* **2021**, *7*, eabk2872, doi:10.1126/sciadv.abk2872.
12. Cao, C.; Kang, H.J.; Singh, I.; Chen, H.; Zhang, C.; Ye, W.; Hayes, B.W.; Liu, J.; Gumpper, R.H.; Bender, B.J.; et al. Structure, Function and Pharmacology of Human Itch GPCRs. *Nature* **2021**, *600*, 170–175, doi:10.1038/s41586-021-04126-6.
13. Hamaguchi, T.; Kawakami, K.; Shinzawa-Itoh, K.; Inoue-Kashino, N.; Itoh, S.; Ifuku, K.; Yamashita, E.; Maeda, K.; Yonekura, K.; Kashino, Y. Structure of the Far-Red Light Utilizing Photosystem I of Acaryochloris Marina. *Nat Commun* **2021**, *12*, 2333, doi:10.1038/s41467-021-22502-8.
14. Lin, S.; Ke, M.; Zhang, Y.; Yan, Z.; Wu, J. Structure of a Mammalian Sperm Cation Channel Complex. *Nature* **2021**, *595*, 746–750, doi:10.1038/s41586-021-03742-6.
15. Qian, P.; Swainsbury, D.J.K.; Croll, T.I.; Salisbury, J.H.; Martin, E.C.; Jackson, P.J.; Hitchcock, A.; Castro-Hartmann, P.; Sader, K.; Hunter, C.N. Cryo-EM Structure of the Monomeric *Rhodobacter Sphaeroides*

RC–LH1 Core Complex at 2.5 Å. *Biochemical Journal* **2021**, *478*, 3775–3790, doi:10.1042/BCJ20210631.
16. Caspy, I.; Fadeeva, M.; Kuhlgert, S.; Borovikova-Sheinker, A.; Klaiman, D.; Masrati, G.; Drepper, F.; Ben-Tal, N.; Hippler, M.; Nelson, N. Structure of Plant Photosystem I-Plastocyanin Complex Reveals Strong Hydrophobic Interactions. *Biochemical Journal* **2021**, *478*, 2371–2384, doi:10.1042/BCJ20210267.
17. Li, J.; Han, L.; Vallese, F.; Ding, Z.; Choi, S.K.; Hong, S.; Luo, Y.; Liu, B.; Chan, C.K.; Tajkhorshid, E.; et al. Cryo-EM Structures of *Escherichia Coli* Cytochrome *Bo* $_3$ Reveal Bound Phospholipids and Ubiquinone-8 in a Dynamic Substrate Binding Site. *Proc. Natl. Acad. Sci. U.S.A.* **2021**, *118*, e2106750118, doi:10.1073/pnas.2106750118.
18. Ge, J.; Elferich, J.; Dehghani-Ghahnaviyeh, S.; Zhao, Z.; Meadows, M.; Von Gersdorff, H.; Tajkhorshid, E.; Gouaux, E. Molecular Mechanism of Prestin Electromotive Signal Amplification. *Cell* **2021**, *184*, 4669-4679.e13, doi:10.1016/j.cell.2021.07.034.
19. Sun, Y.; Wang, J.; Long, T.; Qi, X.; Donnelly, L.; Elghobashi-Meinhardt, N.; Esparza, L.; Cohen, J.C.; Xie, X.-S.; Hobbs, H.H.; et al. Molecular Basis of Cholesterol Efflux via ABCG Subfamily Transporters. *Proc. Natl. Acad. Sci. U.S.A.* **2021**, *118*, e2110483118, doi:10.1073/pnas.2110483118.
20. Wu, Y.; Chen, Z.; Sigworth, F.J.; Canessa, C.M. Structure and Analysis of Nanobody Binding to the Human ASIC1a Ion Channel. *eLife* **2021**, *10*, e67115, doi:10.7554/eLife.67115.
21. Yu, J.; Zhu, H.; Lape, R.; Greiner, T.; Du, J.; Lü, W.; Sivilotti, L.; Gouaux, E. Mechanism of Gating and Partial Agonist Action in the Glycine Receptor. *Cell* **2021**, *184*, 957-968.e21, doi:10.1016/j.cell.2021.01.026.
22. Uchański, T.; Masiulis, S.; Fischer, B.; Kalichuk, V.; López-Sánchez, U.; Zarkadas, E.; Weckener, M.; Sente, A.; Ward, P.; Wohlkönig, A.; et al. Megabodies Expand the Nanobody Toolkit for Protein Structure Determination by Single-Particle Cryo-EM. *Nat Methods* **2021**, *18*, 60–68, doi:10.1038/s41592-020-01001-6.
23. Lyu, M.; Su, C.; Kazura, J.W.; Yu, E.W. Structural Basis of Transport and Inhibition of the *Plasmodium Falciparum* Transporter PfFNT. *EMBO Reports* **2021**, *22*, e51628, doi:10.15252/embr.202051628.
24. Basak, S.; Kumar, A.; Ramsey, S.; Gibbs, E.; Kapoor, A.; Filizola, M.; Chakrapani, S. High-Resolution Structures of Multiple 5-HT3AR-Setron Complexes Reveal a Novel Mechanism of Competitive Inhibition. *eLife* **2020**, *9*, e57870, doi:10.7554/eLife.57870.
25. Qiu, B.; Matthies, D.; Fortea, E.; Yu, Z.; Boudker, O. Cryo-EM Structures of Excitatory Amino Acid Transporter 3 Visualize Coupled Substrate, Sodium, and Proton Binding and Transport. *Sci. Adv.* **2021**, *7*, eabf5814, doi:10.1126/sciadv.abf5814.
26. Wu, D.; Grund, T.N.; Welsch, S.; Mills, D.J.; Michel, M.; Safarian, S.; Michel, H. Structural Basis for Amino Acid Exchange by a Human Heteromeric Amino Acid Transporter. *Proc. Natl. Acad. Sci. U.S.A.* **2020**, *117*, 21281–21287, doi:10.1073/pnas.2008111117.
27. Israeli, H.; Degtjarik, O.; Fierro, F.; Chunilal, V.; Gill, A.K.; Roth, N.J.; Botta, J.; Prabahar, V.; Peleg, Y.; Chan, L.F.; et al. Structure Reveals the Activation Mechanism of the MC4 Receptor to Initiate Satiation Signaling. *Science* **2021**, *372*, 808–814, doi:10.1126/science.abf7958.
28. Hirschi, S.; Kalbermatter, D.; Ucurum, Z.; Lemmin, T.; Fotiadis, D. Cryo-EM Structure and Dynamics of the Green-Light Absorbing Proteorhodopsin. *Nat Commun* **2021**, *12*, 4107, doi:10.1038/s41467-021-24429-6.
29. Sweet, M.E.; Larsen, C.; Zhang, X.; Schlame, M.; Pedersen, B.P.; Stokes, D.L. Structural Basis for Potassium Transport in Prokaryotes by KdpFABC. *Proc. Natl. Acad. Sci. U.S.A.* **2021**, *118*, e2105195118, doi:10.1073/pnas.2105195118.
30. Xu, P.; Huang, S.; Mao, C.; Krumm, B.E.; Zhou, X.E.; Tan, Y.; Huang, X.-P.; Liu, Y.; Shen, D.-D.; Jiang, Y.; et al. Structures of the Human Dopamine D3 Receptor-Gi Complexes. *Molecular Cell* **2021**, *81*, 1147-1159.e4, doi:10.1016/j.molcel.2021.01.003.

31. Qu, C.; Mao, C.; Xiao, P.; Shen, Q.; Zhong, Y.-N.; Yang, F.; Shen, D.-D.; Tao, X.; Zhang, H.; Yan, X.; et al. Ligand Recognition, Unconventional Activation, and G Protein Coupling of the Prostaglandin E $_2$ Receptor EP2 Subtype. *Sci. Adv.* **2021**, *7*, eabf1268, doi:10.1126/sciadv.abf1268.
32. Song, K.; Wei, M.; Guo, W.; Quan, L.; Kang, Y.; Wu, J.-X.; Chen, L. Structural Basis for Human TRPC5 Channel Inhibition by Two Distinct Inhibitors. *eLife* **2021**, *10*, e63429, doi:10.7554/eLife.63429.
33. Wang, W.; Gao, Y.; Tang, Y.; Zhou, X.; Lai, Y.; Zhou, S.; Zhang, Y.; Yang, X.; Liu, F.; Guddat, L.W.; et al. Cryo-EM Structure of Mycobacterial Cytochrome Bd Reveals Two Oxygen Access Channels. *Nat Commun* **2021**, *12*, 4621, doi:10.1038/s41467-021-24924-w.
34. Zhou, X.; Gao, Y.; Wang, W.; Yang, X.; Yang, X.; Liu, F.; Tang, Y.; Lam, S.M.; Shui, G.; Yu, L.; et al. Architecture of the Mycobacterial Succinate Dehydrogenase with a Membrane-Embedded Rieske FeS Cluster. *Proc. Natl. Acad. Sci. U.S.A.* **2021**, *118*, e2022308118, doi:10.1073/pnas.2022308118.
35. Ping, Y.-Q.; Mao, C.; Xiao, P.; Zhao, R.-J.; Jiang, Y.; Yang, Z.; An, W.-T.; Shen, D.-D.; Yang, F.; Zhang, H.; et al. Structures of the Glucocorticoid-Bound Adhesion Receptor GPR97–Go Complex. *Nature* **2021**, *589*, 620–626, doi:10.1038/s41586-020-03083-w.
36. Yang, G.; Zhou, R.; Guo, X.; Yan, C.; Lei, J.; Shi, Y. Structural Basis of γ-Secretase Inhibition and Modulation by Small Molecule Drugs. *Cell* **2021**, *184*, 521-533.e14, doi:10.1016/j.cell.2020.11.049.
37. Zhong, Q.; Zhao, Y.; Ye, F.; Xiao, Z.; Huang, G.; Xu, M.; Zhang, Y.; Zhan, X.; Sun, K.; Wang, Z.; et al. Cryo-EM Structure of Human Wntless in Complex with Wnt3a. *Nat Commun* **2021**, *12*, 4541, doi:10.1038/s41467-021-24731-3.
38. Yan, R.; Li, Y.; Müller, J.; Zhang, Y.; Singer, S.; Xia, L.; Zhong, X.; Gertsch, J.; Altmann, K.-H.; Zhou, Q. Mechanism of Substrate Transport and Inhibition of the Human LAT1-4F2hc Amino Acid Transporter. *Cell Discov* **2021**, *7*, 16, doi:10.1038/s41421-021-00247-4.
39. Zhao, F.; Zhang, C.; Zhou, Q.; Hang, K.; Zou, X.; Chen, Y.; Wu, F.; Rao, Q.; Dai, A.; Yin, W.; et al. Structural Insights into Hormone Recognition by the Human Glucose-Dependent Insulinotropic Polypeptide Receptor. *eLife* **2021**, *10*, e68719, doi:10.7554/eLife.68719.
40. Cong, Z.; Chen, L.-N.; Ma, H.; Zhou, Q.; Zou, X.; Ye, C.; Dai, A.; Liu, Q.; Huang, W.; Sun, X.; et al. Molecular Insights into Ago-Allosteric Modulation of the Human Glucagon-like Peptide-1 Receptor. *Nat Commun* **2021**, *12*, 3763, doi:10.1038/s41467-021-24058-z.
41. Song, D.; Jiao, H.; Liu, Z. Phospholipid Translocation Captured in a Bifunctional Membrane Protein MprF. *Nat Commun* **2021**, *12*, 2927, doi:10.1038/s41467-021-23248-z.
42. Zhou, F.; Ye, C.; Ma, X.; Yin, W.; Croll, T.I.; Zhou, Q.; He, X.; Zhang, X.; Yang, D.; Wang, P.; et al. Molecular Basis of Ligand Recognition and Activation of Human V2 Vasopressin Receptor. *Cell Res* **2021**, *31*, 929–931, doi:10.1038/s41422-021-00480-2.
43. Huang, K.; Zeng, J.; Liu, X.; Jiang, T.; Wang, J. Structure of the Mannose Phosphotransferase System (Man-PTS) Complexed with Microcin E492, a Pore-Forming Bacteriocin. *Cell Discov* **2021**, *7*, 20, doi:10.1038/s41421-021-00253-6.
44. Zhou, S.; Wang, W.; Zhou, X.; Zhang, Y.; Lai, Y.; Tang, Y.; Xu, J.; Li, D.; Lin, J.; Yang, X.; et al. Structure of Mycobacterium Tuberculosis Cytochrome Bcc in Complex with Q203 and TB47, Two Anti-TB Drug Candidates. *eLife* **2021**, *10*, e69418, doi:10.7554/eLife.69418.
45. Xu, P.; Huang, S.; Zhang, H.; Mao, C.; Zhou, X.E.; Cheng, X.; Simon, I.A.; Shen, D.-D.; Yen, H.-Y.; Robinson, C.V.; et al. Structural Insights into the Lipid and Ligand Regulation of Serotonin Receptors. *Nature* **2021**, *592*, 469–473, doi:10.1038/s41586-021-03376-8.
46. Fang, S.; Huang, X.; Zhang, X.; Zhang, M.; Hao, Y.; Guo, H.; Liu, L.-N.; Yu, F.; Zhang, P. Molecular Mechanism Underlying Transport and Allosteric Inhibition of Bicarbonate Transporter SbtA. *Proc. Natl. Acad. Sci. U.S.A.* **2021**, *118*, e2101632118, doi:10.1073/pnas.2101632118.
47. Zhao, Y.; Liu, S.; Zhou, Y.; Zhang, M.; Chen, H.; Eric Xu, H.; Sun, D.; Liu, L.; Tian, C. Structural Basis of Human A7 Nicotinic Acetylcholine Receptor Activation. *Cell Res* **2021**, *31*, 713–716, doi:10.1038/s41422-021-00509-6.

48. Deng, Y.; Kashtoh, H.; Wang, Q.; Zhen, G.; Li, Q.; Tang, L.; Gao, H.; Zhang, C.; Qin, L.; Su, M.; et al. Structure and Activity of SLAC1 Channels for Stomatal Signaling in Leaves. *Proc. Natl. Acad. Sci. U.S.A.* **2021**, *118*, e2015151118, doi:10.1073/pnas.2015151118.
49. Chang, W.-H.; Lin, H.-H.; Tsai, I.-K.; Huang, S.-H.; Chung, S.-C.; Tu, I.-P.; Yu, S.S.-F.; Chan, S.I. Copper Centers in the Cryo-EM Structure of Particulate Methane Monooxygenase Reveal the Catalytic Machinery of Methane Oxidation. *J. Am. Chem. Soc.* **2021**, *143*, 9922–9932, doi:10.1021/jacs.1c04082.
50. Yuan, Y.; Jia, G.; Wu, C.; Wang, W.; Cheng, L.; Li, Q.; Li, Z.; Luo, K.; Yang, S.; Yan, W.; et al. Structures of Signaling Complexes of Lipid Receptors S1PR1 and S1PR5 Reveal Mechanisms of Activation and Drug Recognition. *Cell Res* **2021**, *31*, 1263–1274, doi:10.1038/s41422-021-00566-x.
51. Kise, Y.; Kasuya, G.; Okamoto, H.H.; Yamanouchi, D.; Kobayashi, K.; Kusakizako, T.; Nishizawa, T.; Nakajo, K.; Nureki, O. Structural Basis of Gating Modulation of Kv4 Channel Complexes. *Nature* **2021**, *599*, 158–164, doi:10.1038/s41586-021-03935-z.
52. Wang, X.; Cheng, X.; Zhao, L.; Wang, Y.; Ye, C.; Zou, X.; Dai, A.; Cong, Z.; Chen, J.; Zhou, Q.; et al. Molecular Insights into Differentiated Ligand Recognition of the Human Parathyroid Hormone Receptor 2. *Proc. Natl. Acad. Sci. U.S.A.* **2021**, *118*, e2101279118, doi:10.1073/pnas.2101279118.
53. Zhang, H.; Chen, K.; Tan, Q.; Shao, Q.; Han, S.; Zhang, C.; Yi, C.; Chu, X.; Zhu, Y.; Xu, Y.; et al. Structural Basis for Chemokine Recognition and Receptor Activation of Chemokine Receptor CCR5. *Nat Commun* **2021**, *12*, 4151, doi:10.1038/s41467-021-24438-5.
54. Ma, S.; Chen, Y.; Dai, A.; Yin, W.; Guo, J.; Yang, D.; Zhou, F.; Jiang, Y.; Wang, M.-W.; Xu, H.E. Structural Mechanism of Calcium-Mediated Hormone Recognition and Gβ Interaction by the Human Melanocortin-1 Receptor. *Cell Res* **2021**, *31*, 1061–1071, doi:10.1038/s41422-021-00557-y.
55. Wang, Y.; Guo, S.; Zhuang, Y.; Yun, Y.; Xu, P.; He, X.; Guo, J.; Yin, W.; Xu, H.E.; Xie, X.; et al. Molecular Recognition of an Acyl-Peptide Hormone and Activation of Ghrelin Receptor. *Nat Commun* **2021**, *12*, 5064, doi:10.1038/s41467-021-25364-2.
56. Zhuang, Y.; Xu, P.; Mao, C.; Wang, L.; Krumm, B.; Zhou, X.E.; Huang, S.; Liu, H.; Cheng, X.; Huang, X.-P.; et al. Structural Insights into the Human D1 and D2 Dopamine Receptor Signaling Complexes. *Cell* **2021**, *184*, 931-942.e18, doi:10.1016/j.cell.2021.01.027.
57. Wang, Y.; Niu, Y.; Zhang, Z.; Gable, K.; Gupta, S.D.; Somashekarappa, N.; Han, G.; Zhao, H.; Myasnikov, A.G.; Kalathur, R.C.; et al. Structural Insights into the Regulation of Human Serine Palmitoyltransferase Complexes. *Nat Struct Mol Biol* **2021**, *28*, 240–248, doi:10.1038/s41594-020-00551-9.
58. Moseng, M.A.; Lyu, M.; Pipatpolkai, T.; Glaza, P.; Emerson, C.C.; Stewart, P.L.; Stansfeld, P.J.; Yu, E.W. Cryo-EM Structures of CusA Reveal a Mechanism of Metal-Ion Export. *mBio* **2021**, *12*, e00452-21, doi:10.1128/mBio.00452-21.
59. Zhang, X.; Belousoff, M.J.; Liang, Y.-L.; Danev, R.; Sexton, P.M.; Wootten, D. Structure and Dynamics of Semaglutide- and Taspoglutide-Bound GLP-1R-Gs Complexes. *Cell Reports* **2021**, *36*, 109374, doi:10.1016/j.celrep.2021.109374.
60. Noviello, C.M.; Gharpure, A.; Mukhtasimova, N.; Cabuco, R.; Baxter, L.; Borek, D.; Sine, S.M.; Hibbs, R.E. Structure and Gating Mechanism of the A7 Nicotinic Acetylcholine Receptor. *Cell* **2021**, *184*, 2121-2134.e13, doi:10.1016/j.cell.2021.02.049.
61. Bai, L.; You, Q.; Jain, B.K.; Duan, H.D.; Kovach, A.; Graham, T.R.; Li, H. Transport Mechanism of P4 ATPase Phosphatidylcholine Flippases. *eLife* **2020**, *9*, e62163, doi:10.7554/eLife.62163.
62. Zhang, K.; Julius, D.; Cheng, Y. Structural Snapshots of TRPV1 Reveal Mechanism of Polymodal Functionality. *Cell* **2021**, *184*, 5138-5150.e12, doi:10.1016/j.cell.2021.08.012.
63. Zhang, X.; Johnson, R.M.; Drulyte, I.; Yu, L.; Kotecha, A.; Danev, R.; Wootten, D.; Sexton, P.M.; Belousoff, M.J. Evolving Cryo-EM Structural Approaches for GPCR Drug Discovery. *Structure* **2021**, *29*, 963-974.e6, doi:10.1016/j.str.2021.04.008.

64. Xue, J.; Han, Y.; Zeng, W.; Wang, Y.; Jiang, Y. Structural Mechanisms of Gating and Selectivity of Human Rod CNGA1 Channel. *Neuron* **2021**, *109*, 1302-1313.e4, doi:10.1016/j.neuron.2021.02.007.
65. Wang, L.; Chen, K.; Zhou, M. Structure and Function of an Arabidopsis Thaliana Sulfate Transporter. *Nat Commun* **2021**, *12*, 4455, doi:10.1038/s41467-021-24778-2.
66. Kwon, D.H.; Zhang, F.; Suo, Y.; Bouvette, J.; Borgnia, M.J.; Lee, S.-Y. Heat-Dependent Opening of TRPV1 in the Presence of Capsaicin. *Nat Struct Mol Biol* **2021**, *28*, 554–563, doi:10.1038/s41594-021-00616-3.
67. Gao, Y.; Robertson, M.J.; Rahman, S.N.; Seven, A.B.; Zhang, C.; Meyerowitz, J.G.; Panova, O.; Hannan, F.M.; Thakker, R.V.; Bräuner-Osborne, H.; et al. Asymmetric Activation of the Calcium-Sensing Receptor Homodimer. *Nature* **2021**, *595*, 455–459, doi:10.1038/s41586-021-03691-0.
68. Mobbs, J.I.; Belousoff, M.J.; Harikumar, K.G.; Piper, S.J.; Xu, X.; Furness, S.G.B.; Venugopal, H.; Christopoulos, A.; Danev, R.; Wootten, D.; et al. Structures of the Human Cholecystokinin 1 (CCK1) Receptor Bound to Gs and Gq Mimetic Proteins Provide Insight into Mechanisms of G Protein Selectivity. *PLoS Biol* **2021**, *19*, e3001295, doi:10.1371/journal.pbio.3001295.
69. Schmiege, P.; Fine, M.; Li, X. Atomic Insights into ML-SI3 Mediated Human TRPML1 Inhibition. *Structure* **2021**, *29*, 1295-1302.e3, doi:10.1016/j.str.2021.06.003.
70. Su, C.-C.; Klenotic, P.A.; Cui, M.; Lyu, M.; Morgan, C.E.; Yu, E.W. Structures of the Mycobacterial Membrane Protein MmpL3 Reveal Its Mechanism of Lipid Transport. *PLoS Biol* **2021**, *19*, e3001370, doi:10.1371/journal.pbio.3001370.
71. Sim, S.I.; Von Bülow, S.; Hummer, G.; Park, E. Structural Basis of Polyamine Transport by Human ATP13A2 (PARK9). *Molecular Cell* **2021**, *81*, 4635-4649.e8, doi:10.1016/j.molcel.2021.08.017.
72. Liu, H.; Sun, D.; Myasnikov, A.; Damian, M.; Baneres, J.-L.; Sun, J.; Zhang, C. Structural Basis of Human Ghrelin Receptor Signaling by Ghrelin and the Synthetic Agonist Ibutamoren. *Nat Commun* **2021**, *12*, 6410, doi:10.1038/s41467-021-26735-5.
73. Yang, Y.; Liu, J.; Clarke, B.R.; Seidel, L.; Bolla, J.R.; Ward, P.N.; Zhang, P.; Robinson, C.V.; Whitfield, C.; Naismith, J.H. The Molecular Basis of Regulation of Bacterial Capsule Assembly by Wzc. *Nat Commun* **2021**, *12*, 4349, doi:10.1038/s41467-021-24652-1.
74. Vercellino, I.; Sazanov, L.A. Structure and Assembly of the Mammalian Mitochondrial Supercomplex CIII2CIV. *Nature* **2021**, *598*, 364–367, doi:10.1038/s41586-021-03927-z.
75. Timcenko, M.; Dieudonné, T.; Montigny, C.; Boesen, T.; Lyons, J.A.; Lenoir, G.; Nissen, P. Structural Basis of Substrate-Independent Phosphorylation in a P4-ATPase Lipid Flippase. *Journal of Molecular Biology* **2021**, *433*, 167062, doi:10.1016/j.jmb.2021.167062.
76. Grund, T.N.; Radloff, M.; Wu, D.; Goojani, H.G.; Witte, L.F.; Jösting, W.; Buschmann, S.; Müller, H.; Elamri, I.; Welsch, S.; et al. Mechanistic and Structural Diversity between Cytochrome *Bd* Isoforms of *Escherichia Coli*. *Proc. Natl. Acad. Sci. U.S.A.* **2021**, *118*, e2114013118, doi:10.1073/pnas.2114013118.
77. Harris, A.; Wagner, M.; Du, D.; Raschka, S.; Nentwig, L.-M.; Gohlke, H.; Smits, S.H.J.; Luisi, B.F.; Schmitt, L. Structure and Efflux Mechanism of the Yeast Pleiotropic Drug Resistance Transporter Pdr5. *Nat Commun* **2021**, *12*, 5254, doi:10.1038/s41467-021-25574-8.
78. Ellinghaus, T.L.; Marcellino, T.; Srinivasan, V.; Lill, R.; Kühlbrandt, W. Conformational Changes in the Yeast Mitochondrial ABC Transporter Atm1 during the Transport Cycle. *Sci. Adv.* **2021**, *7*, eabk2392, doi:10.1126/sciadv.abk2392.
79. Raisch, T.; Brockmann, A.; Ebbinghaus-Kintscher, U.; Freigang, J.; Gutbrod, O.; Kubicek, J.; Maertens, B.; Hofnagel, O.; Raunser, S. Small Molecule Modulation of the Drosophila Slo Channel Elucidated by Cryo-EM. *Nat Commun* **2021**, *12*, 7164, doi:10.1038/s41467-021-27435-w.
80. Wang, Q.; Asarnow, D.E.; Ding, K.; Mann, R.K.; Hatakeyama, J.; Zhang, Y.; Ma, Y.; Cheng, Y.; Beachy, P.A. Dispatched Uses Na+ Flux to Power Release of Lipid-Modified Hedgehog. *Nature* **2021**, *599*, 320–324, doi:10.1038/s41586-021-03996-0.

81. Alegre, K.O.; Paknejad, N.; Su, M.; Lou, J.-S.; Huang, J.; Jordan, K.D.; Eng, E.T.; Meyerson, J.R.; Hite, R.K.; Huang, X.-Y. Structural Basis and Mechanism of Activation of Two Different Families of G Proteins by the Same GPCR. *Nat Struct Mol Biol* **2021**, *28*, 936–944, doi:10.1038/s41594-021-00679-2.
82. Neuberger, A.; Nadezhdin, K.D.; Sobolevsky, A.I. Structural Mechanisms of TRPV6 Inhibition by Ruthenium Red and Econazole. *Nat Commun* **2021**, *12*, 6284, doi:10.1038/s41467-021-26608-x.
83. Dickinson, M.S.; Pourmal, S.; Gupta, M.; Bi, M.; Stroud, R.M. Symmetry Reduction in a Hyperpolarization-Activated Homotetrameric Ion Channel. *Biochemistry* **2022**, *61*, 2177–2181, doi:10.1021/acs.biochem.1c00654.
84. Zhu, L.; Zeng, J.; Wang, C.; Wang, J. Structural Basis of Pore Formation in the Mannose Phosphotransferase System by Pediocin PA-1. *Appl Environ Microbiol* **2022**, *88*, e01992-21, doi:10.1128/AEM.01992-21.
85. Zheng, Y.; Liu, H.; Chen, Y.; Dong, S.; Wang, F.; Wang, S.; Li, G.-L.; Shu, Y.; Xu, F. Structural Insights into the Lipid and Ligand Regulation of a Human Neuronal KCNQ Channel. *Neuron* **2022**, *110*, 237-247.e4, doi:10.1016/j.neuron.2021.10.029.
86. Johnson, S.; Furlong, E.J.; Deme, J.C.; Nord, A.L.; Caesar, J.J.E.; Chevance, F.F.V.; Berry, R.M.; Hughes, K.T.; Lea, S.M. Molecular Structure of the Intact Bacterial Flagellar Basal Body. *Nat Microbiol* **2021**, *6*, 712–721, doi:10.1038/s41564-021-00895-y.
87. Takeda, H.; Tsutsumi, A.; Nishizawa, T.; Lindau, C.; Busto, J.V.; Wenz, L.-S.; Ellenrieder, L.; Imai, K.; Straub, S.P.; Mossmann, W.; et al. Mitochondrial Sorting and Assembly Machinery Operates by β-Barrel Switching. *Nature* **2021**, *590*, 163–169, doi:10.1038/s41586-020-03113-7.
88. Tan, J.; Zhang, X.; Wang, X.; Xu, C.; Chang, S.; Wu, H.; Wang, T.; Liang, H.; Gao, H.; Zhou, Y.; et al. Structural Basis of Assembly and Torque Transmission of the Bacterial Flagellar Motor. *Cell* **2021**, *184*, 2665-2679.e19, doi:10.1016/j.cell.2021.03.057.
89. Zhu, H.; Gouaux, E. Architecture and Assembly Mechanism of Native Glycine Receptors. *Nature* **2021**, *599*, 513–517, doi:10.1038/s41586-021-04022-z.
90. Qian, P.; Swainsbury, D.J.K.; Croll, T.I.; Castro-Hartmann, P.; Divitini, G.; Sader, K.; Hunter, C.N. Cryo-EM Structure of the *Rhodobacter Sphaeroides* Light-Harvesting 2 Complex at 2.1 Å. *Biochemistry* **2021**, *60*, 3302–3314, doi:10.1021/acs.biochem.1c00576.
91. Qian, P.; Croll, T.I.; Hitchcock, A.; Jackson, P.J.; Salisbury, J.H.; Castro-Hartmann, P.; Sader, K.; Swainsbury, D.J.K.; Hunter, C.N. Cryo-EM Structure of the Dimeric *Rhodobacter Sphaeroides* RC-LH1 Core Complex at 2.9 Å: The Structural Basis for Dimerisation. *Biochemical Journal* **2021**, *478*, 3923–3937, doi:10.1042/BCJ20210696.
92. Dobson, Z.; Ahad, S.; Vanlandingham, J.; Toporik, H.; Vaughn, N.; Vaughn, M.; Williams, D.; Reppert, M.; Fromme, P.; Mazor, Y. The Structure of Photosystem I from a High-Light-Tolerant Cyanobacteria. *eLife* **2021**, *10*, e67518, doi:10.7554/eLife.67518.
93. Swainsbury, D.J.K.; Qian, P.; Jackson, P.J.; Faries, K.M.; Niedzwiedzki, D.M.; Martin, E.C.; Farmer, D.A.; Malone, L.A.; Thompson, R.F.; Ranson, N.A.; et al. Structures of *Rhodopseudomonas Palustris* RC-LH1 Complexes with Open or Closed Quinone Channels. *Sci. Adv.* **2021**, *7*, eabe2631, doi:10.1126/sciadv.abe2631.
94. Hu, M.; Yang, F.; Huang, Y.; You, X.; Liu, D.; Sun, S.; Sui, S.-F. Structural Insights into the Mechanism of Human NPC1L1-Mediated Cholesterol Uptake. *Sci. Adv.* **2021**, *7*, eabg3188, doi:10.1126/sciadv.abg3188.
95. Pan, X.; Tokutsu, R.; Li, A.; Takizawa, K.; Song, C.; Murata, K.; Yamasaki, T.; Liu, Z.; Minagawa, J.; Li, M. Structural Basis of LhcbM5-Mediated State Transitions in Green Algae. *Nat. Plants* **2021**, *7*, 1119–1131, doi:10.1038/s41477-021-00960-8.

96. Liu, Q.; Yang, D.; Zhuang, Y.; Croll, T.I.; Cai, X.; Dai, A.; He, X.; Duan, J.; Yin, W.; Ye, C.; et al. Ligand Recognition and G-Protein Coupling Selectivity of Cholecystokinin A Receptor. *Nat Chem Biol* **2021**, *17*, 1238–1244, doi:10.1038/s41589-021-00841-3.
97. Baker, M.R.; Fan, G.; Seryshev, A.B.; Agosto, M.A.; Baker, M.L.; Serysheva, I.I. Cryo-EM Structure of Type 1 IP3R Channel in a Lipid Bilayer. *Commun Biol* **2021**, *4*, 625, doi:10.1038/s42003-021-02156-4.
98. Scortecci, J.F.; Molday, L.L.; Curtis, S.B.; Garces, F.A.; Panwar, P.; Van Petegem, F.; Molday, R.S. Cryo-EM Structures of the ABCA4 Importer Reveal Mechanisms Underlying Substrate Binding and Stargardt Disease. *Nat Commun* **2021**, *12*, 5902, doi:10.1038/s41467-021-26161-7.
99. Jiang, Y.; Benz, T.L.; Long, S.B. Substrate and Product Complexes Reveal Mechanisms of Hedgehog Acylation by HHAT. *Science* **2021**, *372*, 1215–1219, doi:10.1126/science.abg4998.
100. Niu, Y.; Tao, X.; Vaisey, G.; Olinares, P.D.B.; Alwaseem, H.; Chait, B.T.; MacKinnon, R. Analysis of the Mechanosensor Channel Functionality of TACAN. *eLife* **2021**, *10*, e71188, doi:10.7554/eLife.71188.
101. Thangaratnarajah, C.; Rheinberger, J.; Paulino, C.; Slotboom, D.J. Insights into the Bilayer-Mediated Toppling Mechanism of a Folate-Specific ECF Transporter by Cryo-EM. *Proc. Natl. Acad. Sci. U.S.A.* **2021**, *118*, e2105014118, doi:10.1073/pnas.2105014118.
102. Graça, A.T.; Hall, M.; Persson, K.; Schröder, W.P. High-Resolution Model of Arabidopsis Photosystem II Reveals the Structural Consequences of Digitonin-Extraction. *Sci Rep* **2021**, *11*, 15534, doi:10.1038/s41598-021-94914-x.
103. Qian, P.; Croll, T.I.; Swainsbury, D.J.K.; Castro-Hartmann, P.; Moriarty, N.W.; Sader, K.; Hunter, C.N. Cryo-EM Structure of the *Rhodospirillum Rubrum* RC–LH1 Complex at 2.5 Å. *Biochemical Journal* **2021**, *478*, 3253–3263, doi:10.1042/BCJ20210511.
104. Gisriel, C.J.; Wang, J.; Liu, J.; Flesher, D.A.; Reiss, K.M.; Huang, H.-L.; Yang, K.R.; Armstrong, W.H.; Gunner, M.R.; Batista, V.S.; et al. High-Resolution Cryo-Electron Microscopy Structure of Photosystem II from the Mesophilic Cyanobacterium, *Synechocystis* Sp. PCC 6803. *Proc. Natl. Acad. Sci. U.S.A.* **2022**, *119*, e2116765118, doi:10.1073/pnas.2116765118.
105. Johnson, R.M.; Zhang, X.; Piper, S.J.; Nettleton, T.J.; Vandekolk, T.H.; Langmead, C.J.; Danev, R.; Sexton, P.M.; Wootten, D. Cryo-EM Structure of the Dual Incretin Receptor Agonist, Peptide-19, in Complex with the Glucagon-like Peptide-1 Receptor. *Biochemical and Biophysical Research Communications* **2021**, *578*, 84–90, doi:10.1016/j.bbrc.2021.09.016.
106. Niu, Y.; Liu, R.; Guan, C.; Zhang, Y.; Chen, Z.; Hoerer, S.; Nar, H.; Chen, L. Structural Basis of Inhibition of the Human SGLT2–MAP17 Glucose Transporter. *Nature* **2022**, *601*, 280–284, doi:10.1038/s41586-021-04212-9.
107. Caspy, I.; Neumann, E.; Fadeeva, M.; Liveanu, V.; Savitsky, A.; Frank, A.; Kalisman, Y.L.; Shkolnisky, Y.; Murik, O.; Treves, H.; et al. Cryo-EM Photosystem I Structure Reveals Adaptation Mechanisms to Extreme High Light in Chlorella Ohadii. *Nat. Plants* **2021**, *7*, 1314–1322, doi:10.1038/s41477-021-00983-1.
108. Caspy, I.; Schwartz, T.; Bayro-Kaiser, V.; Fadeeva, M.; Kessel, A.; Ben-Tal, N.; Nelson, N. Dimeric and High-Resolution Structures of Chlamydomonas Photosystem I from a Temperature-Sensitive Photosystem II Mutant. *Commun Biol* **2021**, *4*, 1380, doi:10.1038/s42003-021-02911-7.
109. Peng, X.; Wang, N.; Zhu, A.; Xu, H.; Li, J.; Zhou, Y.; Wang, C.; Xiao, Q.; Guo, L.; Liu, F.; et al. Structural Characterization of the Plasmodium Falciparum Lactate Transporter PfFNT Alone and in Complex with Antimalarial Compound MMV007839 Reveals Its Inhibition Mechanism. *PLoS Biol* **2021**, *19*, e3001386, doi:10.1371/journal.pbio.3001386.
110. Abe, K.; Yamamoto, K.; Irie, K.; Nishizawa, T.; Oshima, A. Gastric Proton Pump with Two Occluded K+ Engineered with Sodium Pump-Mimetic Mutations. *Nat Commun* **2021**, *12*, 5709, doi:10.1038/s41467-021-26024-1.

111. Yin, Y.-L.; Ye, C.; Zhou, F.; Wang, J.; Yang, D.; Yin, W.; Wang, M.-W.; Xu, H.E.; Jiang, Y. Molecular Basis for Kinin Selectivity and Activation of the Human Bradykinin Receptors. *Nat Struct Mol Biol* **2021**, *28*, 755–761, doi:10.1038/s41594-021-00645-y.
112. Morgan, C.E.; Glaza, P.; Leus, I.V.; Trinh, A.; Su, C.-C.; Cui, M.; Zgurskaya, H.I.; Yu, E.W. Cryoelectron Microscopy Structures of AdeB Illuminate Mechanisms of Simultaneous Binding and Exporting of Substrates. *mBio* **2021**, *12*, e03690-20, doi:10.1128/mBio.03690-20.
113. Wang, L.; Xu, J.; Cao, S.; Sun, D.; Liu, H.; Lu, Q.; Liu, Z.; Du, Y.; Zhang, C. Cryo-EM Structure of the AVP–Vasopressin Receptor 2–Gs Signaling Complex. *Cell Res* **2021**, *31*, 932–934, doi:10.1038/s41422-021-00483-z.
114. Kumar, P.; Cymes, G.D.; Grosman, C. Structure and Function at the Lipid–Protein Interface of a Pentameric Ligand-Gated Ion Channel. *Proc. Natl. Acad. Sci. U.S.A.* **2021**, *118*, e2100164118, doi:10.1073/pnas.2100164118.
115. Zhang, Z.; Morgan, C.E.; Bonomo, R.A.; Yu, E.W. Cryo-EM Determination of Eravacycline-Bound Structures of the Ribosome and the Multidrug Efflux Pump AdeJ of Acinetobacter Baumannii. *mBio* **2021**, *12*, e01031-21, doi:10.1128/mBio.01031-21.
116. Ruan, Z.; Haley, E.; Orozco, I.J.; Sabat, M.; Myers, R.; Roth, R.; Du, J.; Lü, W. Structures of the TRPM5 Channel Elucidate Mechanisms of Activation and Inhibition. *Nat Struct Mol Biol* **2021**, *28*, 604–613, doi:10.1038/s41594-021-00607-4.
117. Safarian, S.; Opel-Reading, H.K.; Wu, D.; Mehdipour, A.R.; Hards, K.; Harold, L.K.; Radloff, M.; Stewart, I.; Welsch, S.; Hummer, G.; et al. The Cryo-EM Structure of the Bd Oxidase from M. Tuberculosis Reveals a Unique Structural Framework and Enables Rational Drug Design to Combat TB. *Nat Commun* **2021**, *12*, 5236, doi:10.1038/s41467-021-25537-z.
118. Xue, J.; Han, Y.; Zeng, W.; Jiang, Y. Structural Mechanisms of Assembly, Permeation, Gating, and Pharmacology of Native Human Rod CNG Channel. *Neuron* **2022**, *110*, 86-95.e5, doi:10.1016/j.neuron.2021.10.006.
119. Gisriel, C.J.; Shen, G.; Ho, M.-Y.; Kurashov, V.; Flesher, D.A.; Wang, J.; Armstrong, W.H.; Golbeck, J.H.; Gunner, M.R.; Vinyard, D.J.; et al. Structure of a Monomeric Photosystem II Core Complex from a Cyanobacterium Acclimated to Far-Red Light Reveals the Functions of Chlorophylls d and f. *Journal of Biological Chemistry* **2022**, *298*, 101424, doi:10.1016/j.jbc.2021.101424.
120. Kato, K.; Miyazaki, N.; Hamaguchi, T.; Nakajima, Y.; Akita, F.; Yonekura, K.; Shen, J.-R. High-Resolution Cryo-EM Structure of Photosystem II Reveals Damage from High-Dose Electron Beams. *Commun Biol* **2021**, *4*, 382, doi:10.1038/s42003-021-01919-3.
121. Yu, H.; Hamaguchi, T.; Nakajima, Y.; Kato, K.; Kawakami, K.; Akita, F.; Yonekura, K.; Shen, J.-R. Cryo-EM Structure of Monomeric Photosystem II at 2.78 Å Resolution Reveals Factors Important for the Formation of Dimer. *Biochimica et Biophysica Acta (BBA) - Bioenergetics* **2021**, *1862*, 148471, doi:10.1016/j.bbabio.2021.148471.
122. Tani, K.; Kanno, R.; Ji, X.-C.; Hall, M.; Yu, L.-J.; Kimura, Y.; Madigan, M.T.; Mizoguchi, A.; Humbel, B.M.; Wang-Otomo, Z.-Y. Cryo-EM Structure of the Photosynthetic LH1-RC Complex from *Rhodospirillum Rubrum*. *Biochemistry* **2021**, *60*, 2483–2491, doi:10.1021/acs.biochem.1c00360.
123. Bracun, L.; Yamagata, A.; Christianson, B.M.; Terada, T.; Canniffe, D.P.; Shirouzu, M.; Liu, L.-N. Cryo-EM Structure of the Photosynthetic RC-LH1-PufX Supercomplex at 2.8-Å Resolution. *Sci. Adv.* **2021**, *7*, eabf8864, doi:10.1126/sciadv.abf8864.
124. Gisriel, C.J.; Flesher, D.A.; Shen, G.; Wang, J.; Ho, M.-Y.; Brudvig, G.W.; Bryant, D.A. Structure of a Photosystem I-Ferredoxin Complex from a Marine Cyanobacterium Provides Insights into Far-Red Light Photoacclimation. *Journal of Biological Chemistry* **2022**, *298*, 101408, doi:10.1016/j.jbc.2021.101408.
125. Tani, K.; Nagashima, K.V.P.; Kanno, R.; Kawamura, S.; Kikuchi, R.; Hall, M.; Yu, L.-J.; Kimura, Y.; Madigan, M.T.; Mizoguchi, A.; et al. A Previously Unrecognized Membrane Protein in the

Rhodobacter Sphaeroides LH1-RC Photocomplex. *Nat Commun* **2021**, *12*, 6300, doi:10.1038/s41467-021-26561-9.
126. Parey, K.; Lasham, J.; Mills, D.J.; Djurabekova, A.; Haapanen, O.; Yoga, E.G.; Xie, H.; Kühlbrandt, W.; Sharma, V.; Vonck, J.; et al. High-Resolution Structure and Dynamics of Mitochondrial Complex I—Insights into the Proton Pumping Mechanism. *Sci. Adv.* **2021**, *7*, eabj3221, doi:10.1126/sciadv.abj3221.
127. Nadezhdin, K.D.; Neuberger, A.; Trofimov, Y.A.; Krylov, N.A.; Sinica, V.; Kupko, N.; Vlachova, V.; Zakharian, E.; Efremov, R.G.; Sobolevsky, A.I. Structural Mechanism of Heat-Induced Opening of a Temperature-Sensitive TRP Channel. *Nat Struct Mol Biol* **2021**, *28*, 564–572, doi:10.1038/s41594-021-00615-4.
128. Xu, J.; Wang, J.; Liu, A.; Zhang, Y.; Gao, X. Structural and Functional Analysis of SsaV Cytoplasmic Domain and Variable Linker States in the Context of the InvA-SsaV Chimeric Protein. *Microbiol Spectr* **2021**, *9*, e01251-21, doi:10.1128/Spectrum.01251-21.
129. Liu, Y.; Xu, P.; Rivara, S.; Liu, C.; Ricci, J.; Ren, X.; Hurley, J.H.; Ablasser, A. Clathrin-Associated AP-1 Controls Termination of STING Signalling. *Nature* **2022**, *610*, 761–767, doi:10.1038/s41586-022-05354-0.
130. Liu, X.; Khara, P.; Baker, M.L.; Christie, P.J.; Hu, B. Structure of a Type IV Secretion System Core Complex Encoded by Multi-Drug Resistance F Plasmids. *Nat Commun* **2022**, *13*, 379, doi:10.1038/s41467-022-28058-5.
131. Zhou, L.; Maldonado, M.; Padavannil, A.; Guo, F.; Letts, J.A. Structures of *Tetrahymena* 's Respiratory Chain Reveal the Diversity of Eukaryotic Core Metabolism. *Science* **2022**, *376*, 831–839, doi:10.1126/science.abn7747.
132. Miotto, M.C.; Weninger, G.; Dridi, H.; Yuan, Q.; Liu, Y.; Wronska, A.; Melville, Z.; Sittenfeld, L.; Reiken, S.; Marks, A.R. Structural Analyses of Human Ryanodine Receptor Type 2 Channels Reveal the Mechanisms for Sudden Cardiac Death and Treatment. *Sci. Adv.* **2022**, *8*, eabo1272, doi:10.1126/sciadv.abo1272.
133. Ye, W.; Zhao, H.; Dai, Y.; Wang, Y.; Lo, Y.; Jan, L.Y.; Lee, C.-H. Activation and Closed-State Inactivation Mechanisms of the Human Voltage-Gated KV4 Channel Complexes. *Molecular Cell* **2022**, *82*, 2427-2442.e4, doi:10.1016/j.molcel.2022.04.032.
134. Kawamoto, A.; Yamada, T.; Yoshida, T.; Sato, Y.; Kato, T.; Tsuge, H. Cryo-EM Structures of the Translocational Binary Toxin Complex CDTa-Bound CDTb-Pore from Clostridioides Difficile. *Nat Commun* **2022**, *13*, 6119, doi:10.1038/s41467-022-33888-4.
135. Jojoa-Cruz, S.; Saotome, K.; Tsui, C.C.A.; Lee, W.-H.; Sansom, M.S.P.; Murthy, S.E.; Patapoutian, A.; Ward, A.B. Structural Insights into the Venus Flytrap Mechanosensitive Ion Channel Flycatcher1. *Nat Commun* **2022**, *13*, 850, doi:10.1038/s41467-022-28511-5.
136. Maloney, F.P.; Kuklewicz, J.; Corey, R.A.; Bi, Y.; Ho, R.; Mateusiak, L.; Pardon, E.; Steyaert, J.; Stansfeld, P.J.; Zimmer, J. Structure, Substrate Recognition and Initiation of Hyaluronan Synthase. *Nature* **2022**, *604*, 195–201, doi:10.1038/s41586-022-04534-2.
137. Chen, H.; Huang, W.; Li, X. Structures of Oxysterol Sensor EBI2/GPR183, a Key Regulator of the Immune Response. *Structure* **2022**, *30*, 1016-1024.e5, doi:10.1016/j.str.2022.04.006.
138. Cao, J.; Belousoff, M.J.; Liang, Y.-L.; Johnson, R.M.; Josephs, T.M.; Fletcher, M.M.; Christopoulos, A.; Hay, D.L.; Danev, R.; Wootten, D.; et al. A Structural Basis for Amylin Receptor Phenotype. *Science* **2022**, *375*, eabm9609, doi:10.1126/science.abm9609.
139. Kato, K.; Hamaguchi, T.; Nagao, R.; Kawakami, K.; Ueno, Y.; Suzuki, T.; Uchida, H.; Murakami, A.; Nakajima, Y.; Yokono, M.; et al. Structural Basis for the Absence of Low-Energy Chlorophylls in a Photosystem I Trimer from Gloeobacter Violaceus. *eLife* **2022**, *11*, e73990, doi:10.7554/eLife.73990.

140. Kasaragod, V.B.; Mortensen, M.; Hardwick, S.W.; Wahid, A.A.; Dorovykh, V.; Chirgadze, D.Y.; Smart, T.G.; Miller, P.S. Mechanisms of Inhibition and Activation of Extrasynaptic Aβ GABAA Receptors. *Nature* **2022**, *602*, 529–533, doi:10.1038/s41586-022-04402-z.
141. Wang, C.; Polovitskaya, M.M.; Delgado, B.D.; Jentsch, T.J.; Long, S.B. Gating Choreography and Mechanism of the Human Proton-Activated Chloride Channel ASOR. *Sci. Adv.* **2022**, *8*, eabm3942, doi:10.1126/sciadv.abm3942.
142. Faust, B.; Billesbølle, C.B.; Suomivuori, C.-M.; Singh, I.; Zhang, K.; Hoppe, N.; Pinto, A.F.M.; Diedrich, J.K.; Muftuoglu, Y.; Szkudlinski, M.W.; et al. Autoantibody Mimicry of Hormone Action at the Thyrotropin Receptor. *Nature* **2022**, doi:10.1038/s41586-022-05159-1.
143. Cao, P.; Bracun, L.; Yamagata, A.; Christianson, B.M.; Negami, T.; Zou, B.; Terada, T.; Canniffe, D.P.; Shirouzu, M.; Li, M.; et al. Structural Basis for the Assembly and Quinone Transport Mechanisms of the Dimeric Photosynthetic RC–LH1 Supercomplex. *Nat Commun* **2022**, *13*, 1977, doi:10.1038/s41467-022-29563-3.
144. Tani, K.; Kanno, R.; Kikuchi, R.; Kawamura, S.; Nagashima, K.V.P.; Hall, M.; Takahashi, A.; Yu, L.-J.; Kimura, Y.; Madigan, M.T.; et al. Asymmetric Structure of the Native Rhodobacter Sphaeroides Dimeric LH1–RC Complex. *Nat Commun* **2022**, *13*, 1904, doi:10.1038/s41467-022-29453-8.
145. Zhu, S.; Sridhar, A.; Teng, J.; Howard, R.J.; Lindahl, E.; Hibbs, R.E. Structural and Dynamic Mechanisms of GABAA Receptor Modulators with Opposing Activities. *Nat Commun* **2022**, *13*, 4582, doi:10.1038/s41467-022-32212-4.
146. Guo, W.; Tang, Q.; Wei, M.; Kang, Y.; Wu, J.-X.; Chen, L. Structural Mechanism of Human TRPC3 and TRPC6 Channel Regulation by Their Intracellular Calcium-Binding Sites. *Neuron* **2022**, *110*, 1023-1035.e5, doi:10.1016/j.neuron.2021.12.023.
147. Zheng, X.; Li, H.; Hu, Z.; Su, D.; Yang, J. Structural and Functional Characterization of an Achromatopsia-Associated Mutation in a Phototransduction Channel. *Commun Biol* **2022**, *5*, 190, doi:10.1038/s42003-022-03120-6.
148. Zarkadas, E.; Pebay-Peyroula, E.; Thompson, M.J.; Schoehn, G.; Uchański, T.; Steyaert, J.; Chipot, C.; Dehez, F.; Baenziger, J.E.; Nury, H. Conformational Transitions and Ligand-Binding to a Muscle-Type Nicotinic Acetylcholine Receptor. *Neuron* **2022**, *110*, 1358-1370.e5, doi:10.1016/j.neuron.2022.01.013.
149. Sente, A.; Desai, R.; Naydenova, K.; Malinauskas, T.; Jounaidi, Y.; Miehling, J.; Zhou, X.; Masiulis, S.; Hardwick, S.W.; Chirgadze, D.Y.; et al. Differential Assembly Diversifies GABAA Receptor Structures and Signalling. *Nature* **2022**, *604*, 190–194, doi:10.1038/s41586-022-04517-3.
150. Rahman, Md.M.; Basta, T.; Teng, J.; Lee, M.; Worrell, B.T.; Stowell, M.H.B.; Hibbs, R.E. Structural Mechanism of Muscle Nicotinic Receptor Desensitization and Block by Curare. *Nat Struct Mol Biol* **2022**, *29*, 386–394, doi:10.1038/s41594-022-00737-3.
151. Fiedorczuk, K.; Chen, J. Mechanism of CFTR Correction by Type I Folding Correctors. *Cell* **2022**, *185*, 158-168.e11, doi:10.1016/j.cell.2021.12.009.
152. Yang, Y.; Wei, M.; Chen, L. Structural Identification of Riluzole-Binding Site on Human TRPC5. *Cell Discov* **2022**, *8*, 67, doi:10.1038/s41421-022-00410-5.
153. Chen, G.; Xu, J.; Inoue, A.; Schmidt, M.F.; Bai, C.; Lu, Q.; Gmeiner, P.; Liu, Z.; Du, Y. Activation and Allosteric Regulation of the Orphan GPR88-Gi1 Signaling Complex. *Nat Commun* **2022**, *13*, 2375, doi:10.1038/s41467-022-30081-5.
154. Xu, Z.; Ikuta, T.; Kawakami, K.; Kise, R.; Qian, Y.; Xia, R.; Sun, M.-X.; Zhang, A.; Guo, C.; Cai, X.-H.; et al. Structural Basis of Sphingosine-1-Phosphate Receptor 1 Activation and Biased Agonism. *Nat Chem Biol* **2022**, *18*, 281–288, doi:10.1038/s41589-021-00930-3.
155. Shen, J.; Zhang, D.; Fu, Y.; Chen, A.; Yang, X.; Zhang, H. Cryo-EM Structures of Human Bradykinin Receptor-Gq Proteins Complexes. *Nat Commun* **2022**, *13*, 714, doi:10.1038/s41467-022-28399-1.

156. Gorski, C.; Riddle, R.; Toporik, H.; Da, Z.; Dobson, Z.; Williams, D.; Mazor, Y. The Structure of the Physcomitrium Patens Photosystem I Reveals a Unique Lhca2 Paralogue Replacing Lhca4. *Nat. Plants* **2022**, *8*, 307–316, doi:10.1038/s41477-022-01099-w.
157. Tillinghast, J.; Drury, S.; Bowser, D.; Benn, A.; Lee, K.P.K. Structural Mechanisms for Gating and Ion Selectivity of the Human Polyamine Transporter ATP13A2. *Molecular Cell* **2021**, *81*, 4650-4662.e4, doi:10.1016/j.molcel.2021.10.002.
158. Catalano, C.; Ben-Hail, D.; Qiu, W.; Blount, P.; Des Georges, A.; Guo, Y. Cryo-EM Structure of Mechanosensitive Channel YnaI Using SMA2000: Challenges and Opportunities. *Membranes* **2021**, *11*, 849, doi:10.3390/membranes11110849.
159. Khera, R.; Mehdipour, A.R.; Bolla, J.R.; Kahnt, J.; Welsch, S.; Ermler, U.; Muenke, C.; Robinson, C.V.; Hummer, G.; Xie, H.; et al. Cryo-EM Structures of Pentameric Autoinducer-2 Exporter from *Escherichia Coli* Reveal Its Transport Mechanism. *The EMBO Journal* **2022**, *41*, e109990, doi:10.15252/embj.2021109990.
160. Moe, A.; Kovalova, T.; Król, S.; Yanofsky, D.J.; Bott, M.; Sjöstrand, D.; Rubinstein, J.L.; Högbom, M.; Brzezinski, P. The Respiratory Supercomplex from C. Glutamicum. *Structure* **2022**, *30*, 338-349.e3, doi:10.1016/j.str.2021.11.008.
161. Brotherton, D.H.; Savva, C.G.; Ragan, T.J.; Dale, N.; Cameron, A.D. Conformational Changes and CO2-Induced Channel Gating in Connexin26. *Structure* **2022**, *30*, 697-706.e4, doi:10.1016/j.str.2022.02.010.
162. Kao, W.-C.; Ortmann De Percin Northumberland, C.; Cheng, T.C.; Ortiz, J.; Durand, A.; Von Loeffelholz, O.; Schilling, O.; Biniossek, M.L.; Klaholz, B.P.; Hunte, C. Structural Basis for Safe and Efficient Energy Conversion in a Respiratory Supercomplex. *Nat Commun* **2022**, *13*, 545, doi:10.1038/s41467-022-28179-x.
163. Reddy, K.D.; Ciftci, D.; Scopelliti, A.J.; Boudker, O. The Archaeal Glutamate Transporter Homologue GltPh Shows Heterogeneous Substrate Binding. *Journal of General Physiology* **2022**, *154*, e202213131, doi:10.1085/jgp.202213131.
164. Zheng, X.; Hu, Z.; Li, H.; Yang, J. Structure of the Human Cone Photoreceptor Cyclic Nucleotide-Gated Channel. *Nat Struct Mol Biol* **2022**, *29*, 40–46, doi:10.1038/s41594-021-00699-y.
165. Cary, B.P.; Deganutti, G.; Zhao, P.; Truong, T.T.; Piper, S.J.; Liu, X.; Belousoff, M.J.; Danev, R.; Sexton, P.M.; Wootten, D.; et al. Structural and Functional Diversity among Agonist-Bound States of the GLP-1 Receptor. *Nat Chem Biol* **2022**, *18*, 256–263, doi:10.1038/s41589-021-00945-w.
166. Koo, C.W.; Tucci, F.J.; He, Y.; Rosenzweig, A.C. Recovery of Particulate Methane Monooxygenase Structure and Activity in a Lipid Bilayer. *Science* **2022**, *375*, 1287–1291, doi:10.1126/science.abm3282.
167. Wang, H.; Hetzer, F.; Huang, W.; Qu, Q.; Meyerowitz, J.; Kaindl, J.; Hübner, H.; Skiniotis, G.; Kobilka, B.K.; Gmeiner, P. Structure-Based Evolution of G Protein-Biased µ-Opioid Receptor Agonists. *Angew Chem Int Ed* **2022**, *61*, e202200269, doi:10.1002/anie.202200269.
168. Barros-Álvarez, X.; Nwokonko, R.M.; Vizurraga, A.; Matzov, D.; He, F.; Papasergi-Scott, M.M.; Robertson, M.J.; Panova, O.; Yardeni, E.H.; Seven, A.B.; et al. The Tethered Peptide Activation Mechanism of Adhesion GPCRs. *Nature* **2022**, *604*, 757–762, doi:10.1038/s41586-022-04575-7.
169. Park, J.; Zuo, H.; Frangaj, A.; Fu, Z.; Yen, L.Y.; Zhang, Z.; Mosyak, L.; Slavkovich, V.N.; Liu, J.; Ray, K.M.; et al. Symmetric Activation and Modulation of the Human Calcium-Sensing Receptor. *Proc. Natl. Acad. Sci. U.S.A.* **2021**, *118*, e2115849118, doi:10.1073/pnas.2115849118.
170. Tan, X.-F.; Bae, C.; Stix, R.; Fernández-Mariño, A.I.; Huffer, K.; Chang, T.-H.; Jiang, J.; Faraldo-Gómez, J.D.; Swartz, K.J. Structure of the Shaker Kv Channel and Mechanism of Slow C-Type Inactivation. *Sci. Adv.* **2022**, *8*, eabm7814, doi:10.1126/sciadv.abm7814.

171. Gan, N.; Han, Y.; Zeng, W.; Wang, Y.; Xue, J.; Jiang, Y. Structural Mechanism of Allosteric Activation of TRPML1 by PI(3,5)P $_2$ and Rapamycin. *Proc. Natl. Acad. Sci. U.S.A.* **2022**, *119*, e2120404119, doi:10.1073/pnas.2120404119.
172. Robertson, M.J.; Meyerowitz, J.G.; Panova, O.; Borrelli, K.; Skiniotis, G. Plasticity in Ligand Recognition at Somatostatin Receptors. *Nat Struct Mol Biol* **2022**, *29*, 210–217, doi:10.1038/s41594-022-00727-5.
173. Fluck, E.C.; Yazici, A.T.; Rohacs, T.; Moiseenkova-Bell, V.Y. Structural Basis of TRPV5 Regulation by Physiological and Pathophysiological Modulators. *Cell Reports* **2022**, *39*, 110737, doi:10.1016/j.celrep.2022.110737.
174. Zhuang, Y.; Wang, L.; Guo, J.; Sun, D.; Wang, Y.; Liu, W.; Xu, H.E.; Zhang, C. Molecular Recognition of Formylpeptides and Diverse Agonists by the Formylpeptide Receptors FPR1 and FPR2. *Nat Commun* **2022**, *13*, 1054, doi:10.1038/s41467-022-28586-0.
175. Keon, K.A.; Benlekbir, S.; Kirsch, S.H.; Müller, R.; Rubinstein, J.L. Cryo-EM of the Yeast V $_O$ Complex Reveals Distinct Binding Sites for Macrolide V-ATPase Inhibitors. *ACS Chem. Biol.* **2022**, *17*, 619–628, doi:10.1021/acschembio.1c00894.
176. Dickinson, M.S.; Lu, J.; Gupta, M.; Marten, I.; Hedrich, R.; Stroud, R.M. Molecular Basis of Multistep Voltage Activation in Plant Two-Pore Channel 1. *Proc. Natl. Acad. Sci. U.S.A.* **2022**, *119*, e2110936119, doi:10.1073/pnas.2110936119.
177. Liu, S.; Paknejad, N.; Zhu, L.; Kihara, Y.; Ray, M.; Chun, J.; Liu, W.; Hite, R.K.; Huang, X.-Y. Differential Activation Mechanisms of Lipid GPCRs by Lysophosphatidic Acid and Sphingosine 1-Phosphate. *Nat Commun* **2022**, *13*, 731, doi:10.1038/s41467-022-28417-2.
178. Wang, N.; He, X.; Zhao, J.; Jiang, H.; Cheng, X.; Xia, Y.; Eric Xu, H.; He, Y. Structural Basis of Leukotriene B4 Receptor 1 Activation. *Nat Commun* **2022**, *13*, 1156, doi:10.1038/s41467-022-28820-9.
179. Shao, Z.; Shen, Q.; Yao, B.; Mao, C.; Chen, L.-N.; Zhang, H.; Shen, D.-D.; Zhang, C.; Li, W.; Du, X.; et al. Identification and Mechanism of G Protein-Biased Ligands for Chemokine Receptor CCR1. *Nat Chem Biol* **2022**, *18*, 264–271, doi:10.1038/s41589-021-00918-z.
180. Xu, Y.; Feng, W.; Zhou, Q.; Liang, A.; Li, J.; Dai, A.; Zhao, F.; Yan, J.; Chen, C.-W.; Li, H.; et al. A Distinctive Ligand Recognition Mechanism by the Human Vasoactive Intestinal Polypeptide Receptor 2. *Nat Commun* **2022**, *13*, 2272, doi:10.1038/s41467-022-30041-z.
181. Tanaka, S.; Morita, M.; Yamagishi, T.; Madapally, H.V.; Hayashida, K.; Khandelia, H.; Gerle, C.; Shigematsu, H.; Oshima, A.; Abe, K. Structural Basis for Binding of Potassium-Competitive Acid Blockers to the Gastric Proton Pump. *J. Med. Chem.* **2022**, *65*, 7843–7853, doi:10.1021/acs.jmedchem.2c00338.
182. Li, Y.; Ding, Y.; Qu, L.; Li, X.; Lai, Q.; Zhao, P.; Gao, Y.; Xiang, C.; Cang, C.; Liu, X.; et al. Structure of the Arabidopsis Guard Cell Anion Channel SLAC1 Suggests Activation Mechanism by Phosphorylation. *Nat Commun* **2022**, *13*, 2511, doi:10.1038/s41467-022-30253-3.
183. Duan, J.; Shen, D.-D.; Zhao, T.; Guo, S.; He, X.; Yin, W.; Xu, P.; Ji, Y.; Chen, L.-N.; Liu, J.; et al. Molecular Basis for Allosteric Agonism and G Protein Subtype Selectivity of Galanin Receptors. *Nat Commun* **2022**, *13*, 1364, doi:10.1038/s41467-022-29072-3.
184. Qu, X.; Qiu, N.; Wang, M.; Zhang, B.; Du, J.; Zhong, Z.; Xu, W.; Chu, X.; Ma, L.; Yi, C.; et al. Structural Basis of Tethered Agonism of the Adhesion GPCRs ADGRD1 and ADGRF1. *Nature* **2022**, *604*, 779–785, doi:10.1038/s41586-022-04580-w.
185. Zhu, Y.; Lin, X.; Zong, X.; Han, S.; Wang, M.; Su, Y.; Ma, L.; Chu, X.; Yi, C.; Zhao, Q.; et al. Structural Basis of FPR2 in Recognition of Aβ42 and Neuroprotection by Humanin. *Nat Commun* **2022**, *13*, 1775, doi:10.1038/s41467-022-29361-x.

186. Qian, P.; Gardiner, A.T.; Šímová, I.; Naydenova, K.; Croll, T.I.; Jackson, P.J.; Nupur; Kloz, M.; Čubáková, P.; Kuzma, M.; et al. 2.4-Å Structure of the Double-Ring *Gemmatimonas Phototrophica* Photosystem. *Sci. Adv.* **2022**, *8*, eabk3139, doi:10.1126/sciadv.abk3139.
187. Caspy, I.; Fadeeva, M.; Mazor, Y.; Nelson, N. Structure of Dunaliella Photosystem II Reveals Conformational Flexibility of Stacked and Unstacked Supercomplexes. *eLife* **2023**, *12*, e81150, doi:10.7554/eLife.81150.
188. Melville, Z.; Dridi, H.; Yuan, Q.; Reiken, S.; Wronska, A.; Liu, Y.; Clarke, O.B.; Marks, A.R. A Drug and ATP Binding Site in Type 1 Ryanodine Receptor. *Structure* **2022**, *30*, 1025-1034.e4, doi:10.1016/j.str.2022.04.010.
189. Vallese, F.; Kim, K.; Yen, L.Y.; Johnston, J.D.; Noble, A.J.; Calì, T.; Clarke, O.B. Architecture of the Human Erythrocyte Ankyrin-1 Complex. *Nat Struct Mol Biol* **2022**, *29*, 706–718, doi:10.1038/s41594-022-00792-w.
190. Nagao, R.; Kato, K.; Kumazawa, M.; Ifuku, K.; Yokono, M.; Suzuki, T.; Dohmae, N.; Akita, F.; Akimoto, S.; Miyazaki, N.; et al. Structural Basis for Different Types of Hetero-Tetrameric Light-Harvesting Complexes in a Diatom PSII-FCPII Supercomplex. *Nat Commun* **2022**, *13*, 1764, doi:10.1038/s41467-022-29294-5.
191. Farci, D.; Haniewicz, P.; De Sanctis, D.; Iesu, L.; Kereïche, S.; Winterhalter, M.; Piano, D. The Cryo-EM Structure of the S-Layer Deinoxanthin-Binding Complex of Deinococcus Radiodurans Informs Properties of Its Environmental Interactions. *Journal of Biological Chemistry* **2022**, *298*, 102031, doi:10.1016/j.jbc.2022.102031.
192. Guo, Y.; Zhang, Y.; Yan, R.; Huang, B.; Ye, F.; Wu, L.; Chi, X.; Shi, Y.; Zhou, Q. Cryo-EM Structures of Recombinant Human Sodium-Potassium Pump Determined in Three Different States. *Nat Commun* **2022**, *13*, 3957, doi:10.1038/s41467-022-31602-y.
193. Chen, G.; Wang, X.; Liao, Q.; Ge, Y.; Jiao, H.; Chen, Q.; Liu, Y.; Lyu, W.; Zhu, L.; Van Zundert, G.C.P.; et al. Structural Basis for Recognition of N-Formyl Peptides as Pathogen-Associated Molecular Patterns. *Nat Commun* **2022**, *13*, 5232, doi:10.1038/s41467-022-32822-y.
194. Li, J.; Zheng, W.; Gu, M.; Han, L.; Luo, Y.; Yu, K.; Sun, M.; Zong, Y.; Ma, X.; Liu, B.; et al. Structures of the CcmABCD Heme Release Complex at Multiple States. *Nat Commun* **2022**, *13*, 6422, doi:10.1038/s41467-022-34136-5.
195. Lu, Y.; Yu, M.; Jia, Y.; Yang, F.; Zhang, Y.; Xu, X.; Li, X.; Yang, F.; Lei, J.; Wang, Y.; et al. Structural Basis for the Activity Regulation of a Potassium Channel AKT1 from Arabidopsis. *Nat Commun* **2022**, *13*, 5682, doi:10.1038/s41467-022-33420-8.
196. Kumar, A.; Kindig, K.; Rao, S.; Zaki, A.-M.; Basak, S.; Sansom, M.S.P.; Biggin, P.C.; Chakrapani, S. Structural Basis for Cannabinoid-Induced Potentiation of Alpha1-Glycine Receptors in Lipid Nanodiscs. *Nat Commun* **2022**, *13*, 4862, doi:10.1038/s41467-022-32594-5.
197. Kravchuk, V.; Petrova, O.; Kampjut, D.; Wojciechowska-Bason, A.; Breese, Z.; Sazanov, L. A Universal Coupling Mechanism of Respiratory Complex I. *Nature* **2022**, *609*, 808–814, doi:10.1038/s41586-022-05199-7.
198. Galazzo, L.; Meier, G.; Januliene, D.; Parey, K.; De Vecchis, D.; Striednig, B.; Hilbi, H.; Schäfer, L.V.; Kuprov, I.; Moeller, A.; et al. The ABC Transporter MsbA Adopts the Wide Inward-Open Conformation in *E. Coli* Cells. *Sci. Adv.* **2022**, *8*, eabn6845, doi:10.1126/sciadv.abn6845.
199. Coupland, C.E.; Andrei, S.A.; Ansell, T.B.; Carrique, L.; Kumar, P.; Sefer, L.; Schwab, R.A.; Byrne, E.F.X.; Pardon, E.; Steyaert, J.; et al. Structure, Mechanism, and Inhibition of Hedgehog Acyltransferase. *Molecular Cell* **2021**, *81*, 5025-5038.e10, doi:10.1016/j.molcel.2021.11.018.
200. Velazhahan, V.; Ma, N.; Vaidehi, N.; Tate, C.G. Activation Mechanism of the Class D Fungal GPCR Dimer Ste2. *Nature* **2022**, *603*, 743–748, doi:10.1038/s41586-022-04498-3.
201. Chung, I.; Wright, J.J.; Bridges, H.R.; Ivanov, B.S.; Biner, O.; Pereira, C.S.; Arantes, G.M.; Hirst, J. Cryo-EM Structures Define Ubiquinone-10 Binding to Mitochondrial Complex I and Conformational

Transitions Accompanying Q-Site Occupancy. *Nat Commun* **2022**, *13*, 2758, doi:10.1038/s41467-022-30506-1.
202. Chou, T.-H.; Epstein, M.; Michalski, K.; Fine, E.; Biggin, P.C.; Furukawa, H. Structural Insights into Binding of Therapeutic Channel Blockers in NMDA Receptors. *Nat Struct Mol Biol* **2022**, *29*, 507–518, doi:10.1038/s41594-022-00772-0.
203. Zhao, H.; Lee, J.; Chen, J. The Hemolysin A Secretion System Is a Multi-Engine Pump Containing Three ABC Transporters. *Cell* **2022**, *185*, 3329-3340.e13, doi:10.1016/j.cell.2022.07.017.
204. Cao, C.; Barros-Álvarez, X.; Zhang, S.; Kim, K.; Dämgen, M.A.; Panova, O.; Suomivuori, C.-M.; Fay, J.F.; Zhong, X.; Krumm, B.E.; et al. Signaling Snapshots of a Serotonin Receptor Activated by the Prototypical Psychedelic LSD. *Neuron* **2022**, *110*, 3154-3167.e7, doi:10.1016/j.neuron.2022.08.006.
205. Schmidpeter, P.A.M.; Wu, D.; Rheinberger, J.; Riegelhaupt, P.M.; Tang, H.; Robinson, C.V.; Nimigean, C.M. Anionic Lipids Unlock the Gates of Select Ion Channels in the Pacemaker Family. *Nat Struct Mol Biol* **2022**, *29*, 1092–1100, doi:10.1038/s41594-022-00851-2.
206. Zhao, L.-H.; Lin, J.; Ji, S.-Y.; Zhou, X.E.; Mao, C.; Shen, D.-D.; He, X.; Xiao, P.; Sun, J.; Melcher, K.; et al. Structure Insights into Selective Coupling of G Protein Subtypes by a Class B G Protein-Coupled Receptor. *Nat Commun* **2022**, *13*, 6670, doi:10.1038/s41467-022-33851-3.
207. Robertson, M.J.; Papasergi-Scott, M.M.; He, F.; Seven, A.B.; Meyerowitz, J.G.; Panova, O.; Peroto, M.C.; Che, T.; Skiniotis, G. Structure Determination of Inactive-State GPCRs with a Universal Nanobody. *Nat Struct Mol Biol* **2022**, *29*, 1188–1195, doi:10.1038/s41594-022-00859-8.
208. Zhang, S.; Chen, H.; Zhang, C.; Yang, Y.; Popov, P.; Liu, J.; Krumm, B.E.; Cao, C.; Kim, K.; Xiong, Y.; et al. Inactive and Active State Structures Template Selective Tools for the Human 5-HT5A Receptor. *Nat Struct Mol Biol* **2022**, *29*, 677–687, doi:10.1038/s41594-022-00796-6.
209. Oh, S.; Marinelli, F.; Zhou, W.; Lee, J.; Choi, H.J.; Kim, M.; Faraldo-Gómez, J.D.; Hite, R.K. Differential Ion Dehydration Energetics Explains Selectivity in the Non-Canonical Lysosomal K+ Channel TMEM175. *eLife* **2022**, *11*, e75122, doi:10.7554/eLife.75122.
210. Liu, Y.; Qi, X.; Donnelly, L.; Elghobashi-Meinhardt, N.; Long, T.; Zhou, R.W.; Sun, Y.; Wang, B.; Li, X. Mechanisms and Inhibition of Porcupine-Mediated Wnt Acylation. *Nature* **2022**, *607*, 816–822, doi:10.1038/s41586-022-04952-2.
211. Gu, J.; Liu, T.; Guo, R.; Zhang, L.; Yang, M. The Coupling Mechanism of Mammalian Mitochondrial Complex I. *Nat Struct Mol Biol* **2022**, *29*, 172–182, doi:10.1038/s41594-022-00722-w.
212. Qin, J.; Cai, Y.; Xu, Z.; Ming, Q.; Ji, S.-Y.; Wu, C.; Zhang, H.; Mao, C.; Shen, D.-D.; Hirata, K.; et al. Molecular Mechanism of Agonism and Inverse Agonism in Ghrelin Receptor. *Nat Commun* **2022**, *13*, 300, doi:10.1038/s41467-022-27975-9.
213. Wang, M.; Wu, J.-X.; Ding, D.; Chen, L. Structural Insights into the Mechanism of Pancreatic KATP Channel Regulation by Nucleotides. *Nat Commun* **2022**, *13*, 2770, doi:10.1038/s41467-022-30430-4.
214. Ma, D.; Zhao, C.; Wang, X.; Li, X.; Zha, Y.; Zhang, Y.; Fu, G.; Liang, P.; Guo, J.; Lai, D. Structural Basis for the Gating Modulation of Kv4.3 by Auxiliary Subunits. *Cell Res* **2022**, *32*, 411–414, doi:10.1038/s41422-021-00608-4.
215. Huang, X.; Jin, X.; Huang, G.; Huang, J.; Wu, T.; Li, Z.; Chen, J.; Kong, F.; Pan, X.; Yan, N. Structural Basis for High-Voltage Activation and Subtype-Specific Inhibition of Human Na $_v$ 1.8. *Proc. Natl. Acad. Sci. U.S.A.* **2022**, *119*, e2208211119, doi:10.1073/pnas.2208211119.
216. Su, N.; Zhu, A.; Tao, X.; Ding, Z.J.; Chang, S.; Ye, F.; Zhang, Y.; Zhao, C.; Chen, Q.; Wang, J.; et al. Structures and Mechanisms of the Arabidopsis Auxin Transporter PIN3. *Nature* **2022**, *609*, 616–621, doi:10.1038/s41586-022-05142-w.
217. Zhu, X.; Qian, Y.; Li, X.; Xu, Z.; Xia, R.; Wang, N.; Liang, J.; Yin, H.; Zhang, A.; Guo, C.; et al. Structural Basis of Adhesion GPCR GPR110 Activation by Stalk Peptide and G-Proteins Coupling. *Nat Commun* **2022**, *13*, 5513, doi:10.1038/s41467-022-33173-4.

218. Qian, Y.; Ma, Z.; Liu, C.; Li, X.; Zhu, X.; Wang, N.; Xu, Z.; Xia, R.; Liang, J.; Duan, Y.; et al. Structural Insights into Adhesion GPCR ADGRL3 Activation and Gq, Gs, Gi, and G12 Coupling. *Molecular Cell* **2022**, *82*, 4340-4352.e6, doi:10.1016/j.molcel.2022.10.009.
219. Cong, Z.; Zhou, Q.; Li, Y.; Chen, L.-N.; Zhang, Z.-C.; Liang, A.; Liu, Q.; Wu, X.; Dai, A.; Xia, T.; et al. Structural Basis of Peptidomimetic Agonism Revealed by Small-Molecule GLP-1R Agonists Boc5 and WB4-24. *Proc. Natl. Acad. Sci. U.S.A.* **2022**, *119*, e2200155119, doi:10.1073/pnas.2200155119.
220. Shao, Z.; Tan, Y.; Shen, Q.; Hou, L.; Yao, B.; Qin, J.; Xu, P.; Mao, C.; Chen, L.-N.; Zhang, H.; et al. Molecular Insights into Ligand Recognition and Activation of Chemokine Receptors CCR2 and CCR3. *Cell Discov* **2022**, *8*, 44, doi:10.1038/s41421-022-00403-4.
221. Bo, Q.; Yang, F.; Li, Y.; Meng, X.; Zhang, H.; Zhou, Y.; Ling, S.; Sun, D.; Lv, P.; Liu, L.; et al. Structural Insights into the Activation of Somatostatin Receptor 2 by Cyclic SST Analogues. *Cell Discov* **2022**, *8*, 47, doi:10.1038/s41421-022-00405-2.
222. Su, N.; Zhen, W.; Zhang, H.; Xu, L.; Jin, Y.; Chen, X.; Zhao, C.; Wang, Q.; Wang, X.; Li, S.; et al. Structural Mechanisms of TRPV2 Modulation by Endogenous and Exogenous Ligands. *Nat Chem Biol* **2023**, *19*, 72–80, doi:10.1038/s41589-022-01139-8.
223. Fan, J.; Hu, L.; Yue, Z.; Liao, D.; Guo, F.; Ke, H.; Jiang, D.; Yang, Y.; Lei, X. Structural Basis of TRPV3 Inhibition by an Antagonist. *Nat Chem Biol* **2023**, *19*, 81–90, doi:10.1038/s41589-022-01166-5.
224. Kishikawa, J.; Ishikawa, M.; Masuya, T.; Murai, M.; Kitazumi, Y.; Butler, N.L.; Kato, T.; Barquera, B.; Miyoshi, H. Cryo-EM Structures of Na+-Pumping NADH-Ubiquinone Oxidoreductase from Vibrio Cholerae. *Nat Commun* **2022**, *13*, 4082, doi:10.1038/s41467-022-31718-1.
225. Zhao, W.; Han, S.; Qiu, N.; Feng, W.; Lu, M.; Zhang, W.; Wang, M.; Zhou, Q.; Chen, S.; Xu, W.; et al. Structural Insights into Ligand Recognition and Selectivity of Somatostatin Receptors. *Cell Res* **2022**, *32*, 761–772, doi:10.1038/s41422-022-00679-x.
226. Duan, J.; Xu, P.; Luan, X.; Ji, Y.; He, X.; Song, N.; Yuan, Q.; Jin, Y.; Cheng, X.; Jiang, H.; et al. Hormone- and Antibody-Mediated Activation of the Thyrotropin Receptor. *Nature* **2022**, *609*, 854–859, doi:10.1038/s41586-022-05173-3.
227. Xu, P.; Huang, S.; Guo, S.; Yun, Y.; Cheng, X.; He, X.; Cai, P.; Lan, Y.; Zhou, H.; Jiang, H.; et al. Structural Identification of Lysophosphatidylcholines as Activating Ligands for Orphan Receptor GPR119. *Nat Struct Mol Biol* **2022**, *29*, 863–870, doi:10.1038/s41594-022-00816-5.
228. Steinhilper, R.; Höff, G.; Heider, J.; Murphy, B.J. Structure of the Membrane-Bound Formate Hydrogenlyase Complex from Escherichia Coli. *Nat Commun* **2022**, *13*, 5395, doi:10.1038/s41467-022-32831-x.
229. Neumann, C.; Rosenbæk, L.L.; Flygaard, R.K.; Habeck, M.; Karlsen, J.L.; Wang, Y.; Lindorff-Larsen, K.; Gad, H.H.; Hartmann, R.; Lyons, J.A.; et al. Cryo-EM Structure of the Human NKCC1 Transporter Reveals Mechanisms of Ion Coupling and Specificity. *The EMBO Journal* **2022**, *41*, e110169, doi:10.15252/embj.2021110169.
230. Owji, A.P.; Wang, J.; Kittredge, A.; Clark, Z.; Zhang, Y.; Hendrickson, W.A.; Yang, T. Structures and Gating Mechanisms of Human Bestrophin Anion Channels. *Nat Commun* **2022**, *13*, 3836, doi:10.1038/s41467-022-31437-7.
231. Gumpper, R.H.; Fay, J.F.; Roth, B.L. Molecular Insights into the Regulation of Constitutive Activity by RNA Editing of 5HT2C Serotonin Receptors. *Cell Reports* **2022**, *40*, 111211, doi:10.1016/j.celrep.2022.111211.
232. Zhuang, Y.; Wang, Y.; He, B.; He, X.; Zhou, X.E.; Guo, S.; Rao, Q.; Yang, J.; Liu, J.; Zhou, Q.; et al. Molecular Recognition of Morphine and Fentanyl by the Human μ-Opioid Receptor. *Cell* **2022**, *185*, 4361-4375.e19, doi:10.1016/j.cell.2022.09.041.
233. Xu, J.; He, Y.; Wu, X.; Li, L. Conformational Changes of a Phosphatidylcholine Flippase in Lipid Membranes. *Cell Reports* **2022**, *38*, 110518, doi:10.1016/j.celrep.2022.110518.

234. Ren, Y.; Li, Y.; Wang, Y.; Wen, T.; Lu, X.; Chang, S.; Zhang, X.; Shen, Y.; Yang, X. Cryo-EM Structure of the Heptameric Calcium Homeostasis Modulator 1 Channel. *Journal of Biological Chemistry* **2022**, *298*, 101838, doi:10.1016/j.jbc.2022.101838.
235. Hagino, T.; Kato, T.; Kasuya, G.; Kobayashi, K.; Kusakizako, T.; Hamamoto, S.; Sobajima, T.; Fujiwara, Y.; Yamashita, K.; Kawasaki, H.; et al. Cryo-EM Structures of Thylakoid-Located Voltage-Dependent Chloride Channel VCCN1. *Nat Commun* **2022**, *13*, 2505, doi:10.1038/s41467-022-30292-w.
236. Lee, Y.; Wiriyasermkul, P.; Kongpracha, P.; Moriyama, S.; Mills, D.J.; Kühlbrandt, W.; Nagamori, S. Ca2+-Mediated Higher-Order Assembly of Heterodimers in Amino Acid Transport System B0,+ Biogenesis and Cystinuria. *Nat Commun* **2022**, *13*, 2708, doi:10.1038/s41467-022-30293-9.
237. Sun, B.; Willard, F.S.; Feng, D.; Alsina-Fernandez, J.; Chen, Q.; Vieth, M.; Ho, J.D.; Showalter, A.D.; Stutsman, C.; Ding, L.; et al. Structural Determinants of Dual Incretin Receptor Agonism by Tirzepatide. *Proc. Natl. Acad. Sci. U.S.A.* **2022**, *119*, e2116506119, doi:10.1073/pnas.2116506119.
238. Falzone, M.E.; Feng, Z.; Alvarenga, O.E.; Pan, Y.; Lee, B.; Cheng, X.; Fortea, E.; Scheuring, S.; Accardi, A. TMEM16 Scramblases Thin the Membrane to Enable Lipid Scrambling. *Nat Commun* **2022**, *13*, 2604, doi:10.1038/s41467-022-30300-z.
239. Morgan, C.E.; Zhang, Z.; Bonomo, R.A.; Yu, E.W. An Analysis of the Novel Fluorocycline TP-6076 Bound to Both the Ribosome and Multidrug Efflux Pump AdeJ from Acinetobacter Baumannii. *mBio* **2022**, *13*, e03732-21, doi:10.1128/mbio.03732-21.
240. Meyerowitz, J.G.; Robertson, M.J.; Barros-Álvarez, X.; Panova, O.; Nwokonko, R.M.; Gao, Y.; Skiniotis, G. The Oxytocin Signaling Complex Reveals a Molecular Switch for Cation Dependence. *Nat Struct Mol Biol* **2022**, *29*, 274–281, doi:10.1038/s41594-022-00728-4.
241. Noland, C.L.; Chua, H.C.; Kschonsak, M.; Heusser, S.A.; Braun, N.; Chang, T.; Tam, C.; Tang, J.; Arthur, C.P.; Ciferri, C.; et al. Structure-Guided Unlocking of NaX Reveals a Non-Selective Tetrodotoxin-Sensitive Cation Channel. *Nat Commun* **2022**, *13*, 1416, doi:10.1038/s41467-022-28984-4.
242. Cong, Z.; Zhou, F.; Zhang, C.; Zou, X.; Zhang, H.; Wang, Y.; Zhou, Q.; Cai, X.; Liu, Q.; Li, J.; et al. Constitutive Signal Bias Mediated by the Human GHRHR Splice Variant 1. *Proc. Natl. Acad. Sci. U.S.A.* **2021**, *118*, e2106606118, doi:10.1073/pnas.2106606118.
243. You, C.; Zhang, Y.; Xu, P.; Huang, S.; Yin, W.; Eric Xu, H.; Jiang, Y. Structural Insights into the Peptide Selectivity and Activation of Human Neuromedin U Receptors. *Nat Commun* **2022**, *13*, 2045, doi:10.1038/s41467-022-29683-w.
244. Xu, Y.; Jia, G.; Li, T.; Zhou, Z.; Luo, Y.; Chao, Y.; Bao, J.; Su, Z.; Qu, Q.; Li, D. Molecular Insights into Biogenesis of Glycosylphosphatidylinositol Anchor Proteins. *Nat Commun* **2022**, *13*, 2617, doi:10.1038/s41467-022-30250-6.
245. Xiao, P.; Guo, S.; Wen, X.; He, Q.-T.; Lin, H.; Huang, S.-M.; Gou, L.; Zhang, C.; Yang, Z.; Zhong, Y.-N.; et al. Tethered Peptide Activation Mechanism of the Adhesion GPCRs ADGRG2 and ADGRG4. *Nature* **2022**, *604*, 771–778, doi:10.1038/s41586-022-04590-8.
246. Qu, Q.; Huang, W.; Aydin, D.; Paggi, J.M.; Seven, A.B.; Wang, H.; Chakraborty, S.; Che, T.; DiBerto, J.F.; Robertson, M.J.; et al. Insights into Distinct Signaling Profiles of the µOR Activated by Diverse Agonists. *Nat Chem Biol* **2023**, *19*, 423–430, doi:10.1038/s41589-022-01208-y.
247. Su, J.; Liu, D.; Yang, F.; Zuo, M.-Q.; Li, C.; Dong, M.-Q.; Sun, S.; Sui, S.-F. Structural Basis of Tom20 and Tom22 Cytosolic Domains as the Human TOM Complex Receptors. *Proc. Natl. Acad. Sci. U.S.A.* **2022**, *119*, e2200158119, doi:10.1073/pnas.2200158119.
248. Dong, S.; Huang, G.; Wang, C.; Wang, J.; Sui, S.-F.; Qin, X. Structure of the Acidobacteria Homodimeric Reaction Center Bound with Cytochrome c. *Nat Commun* **2022**, *13*, 7745, doi:10.1038/s41467-022-35460-6.

249. Qian, Y.; Wang, J.; Yang, L.; Liu, Y.; Wang, L.; Liu, W.; Lin, Y.; Yang, H.; Ma, L.; Ye, S.; et al. Activation and Signaling Mechanism Revealed by GPR119-Gs Complex Structures. *Nat Commun* **2022**, *13*, 7033, doi:10.1038/s41467-022-34696-6.
250. Nishida, Y.; Yanagisawa, S.; Morita, R.; Shigematsu, H.; Shinzawa-Itoh, K.; Yuki, H.; Ogasawara, S.; Shimuta, K.; Iwamoto, T.; Nakabayashi, C.; et al. Identifying Antibiotics Based on Structural Differences in the Conserved Allostery from Mitochondrial Heme-Copper Oxidases. *Nat Commun* **2022**, *13*, 7591, doi:10.1038/s41467-022-34771-y.
251. Ma, D.; Zhong, L.; Yan, Z.; Yao, J.; Zhang, Y.; Ye, F.; Huang, Y.; Lai, D.; Yang, W.; Hou, P.; et al. Structural Mechanisms for the Activation of Human Cardiac KCNQ1 Channel by Electro-Mechanical Coupling Enhancers. *Proc. Natl. Acad. Sci. U.S.A.* **2022**, *119*, e2207067119, doi:10.1073/pnas.2207067119.
252. Arndt, M.; Alvadia, C.; Straub, M.S.; Clerico Mosina, V.; Paulino, C.; Dutzler, R. Structural Basis for the Activation of the Lipid Scramblase TMEM16F. *Nat Commun* **2022**, *13*, 6692, doi:10.1038/s41467-022-34497-x.
253. Oh, S.; Stix, R.; Zhou, W.; Faraldo-Gómez, J.D.; Hite, R.K. Mechanism of 4-Aminopyridine Inhibition of the Lysosomal Channel TMEM175. *Proc. Natl. Acad. Sci. U.S.A.* **2022**, *119*, e2208882119, doi:10.1073/pnas.2208882119.
254. Piper, S.J.; Deganutti, G.; Lu, J.; Zhao, P.; Liang, Y.-L.; Lu, Y.; Fletcher, M.M.; Hossain, M.A.; Christopoulos, A.; Reynolds, C.A.; et al. Understanding VPAC Receptor Family Peptide Binding and Selectivity. *Nat Commun* **2022**, *13*, 7013, doi:10.1038/s41467-022-34629-3.
255. Zhang, S.; Gumpper, R.H.; Huang, X.-P.; Liu, Y.; Krumm, B.E.; Cao, C.; Fay, J.F.; Roth, B.L. Molecular Basis for Selective Activation of DREADD-Based Chemogenetics. *Nature* **2022**, *612*, 354–362, doi:10.1038/s41586-022-05489-0.
256. Fiedorczuk, K.; Chen, J. Molecular Structures Reveal Synergistic Rescue of Δ508 CFTR by Trikafta Modulators. *Science* **2022**, *378*, 284–290, doi:10.1126/science.ade2216.
257. Macé, K.; Vadakkepat, A.K.; Redzej, A.; Lukoyanova, N.; Oomen, C.; Braun, N.; Ukleja, M.; Lu, F.; Costa, T.R.D.; Orlova, E.V.; et al. Cryo-EM Structure of a Type IV Secretion System. *Nature* **2022**, *607*, 191–196, doi:10.1038/s41586-022-04859-y.
258. Botte, M.; Huber, S.; Bucher, D.; Klint, J.K.; Rodríguez, D.; Tagmose, L.; Chami, M.; Cheng, R.; Hennig, M.; Abdul Rahman, W. Apo and Ligand-Bound High Resolution Cryo-EM Structures of the Human Kv3.1 Channel Reveal a Novel Binding Site for Positive Modulators. *PNAS Nexus* **2022**, *1*, pgac083, doi:10.1093/pnasnexus/pgac083.
259. Li, P.; Hendricks, A.L.; Wang, Y.; Villones, R.L.E.; Lindkvist-Petersson, K.; Meloni, G.; Cowan, J.A.; Wang, K.; Gourdon, P. Structures of Atm1 Provide Insight into [2Fe-2S] Cluster Export from Mitochondria. *Nat Commun* **2022**, *13*, 4339, doi:10.1038/s41467-022-32006-8.
260. Lee, Y.; Haapanen, O.; Altmeyer, A.; Kühlbrandt, W.; Sharma, V.; Zickermann, V. Ion Transfer Mechanisms in Mrp-Type Antiporters from High Resolution cryoEM and Molecular Dynamics Simulations. *Nat Commun* **2022**, *13*, 6091, doi:10.1038/s41467-022-33640-y.
261. Proctor, M.S.; Malone, L.A.; Farmer, D.A.; Swainsbury, D.J.K.; Hawkings, F.R.; Pastorelli, F.; Emrich-Mills, T.Z.; Siebert, C.A.; Hunter, C.N.; Johnson, M.P.; et al. Cryo-EM Structures of the *Synechocystis* Sp. PCC 6803 Cytochrome *b* 6 *f* Complex with and without the Regulatory PetP Subunit. *Biochemical Journal* **2022**, *479*, 1487–1503, doi:10.1042/BCJ20220124.
262. Zhao, Y.; Roy, K.; Vidossich, P.; Cancedda, L.; De Vivo, M.; Forbush, B.; Cao, E. Structural Basis for Inhibition of the Cation-Chloride Cotransporter NKCC1 by the Diuretic Drug Bumetanide. *Nat Commun* **2022**, *13*, 2747, doi:10.1038/s41467-022-30407-3.
263. Selvakumar, P.; Fernández-Mariño, A.I.; Khanra, N.; He, C.; Paquette, A.J.; Wang, B.; Huang, R.; Smider, V.V.; Rice, W.J.; Swartz, K.J.; et al. Structures of the T Cell Potassium Channel Kv1.3 with Immunoglobulin Modulators. *Nat Commun* **2022**, *13*, 3854, doi:10.1038/s41467-022-31285-5.

264. Ren, Z.; Chhetri, A.; Guan, Z.; Suo, Y.; Yokoyama, K.; Lee, S.-Y. Structural Basis for Inhibition and Regulation of a Chitin Synthase from Candida Albicans. *Nat Struct Mol Biol* **2022**, *29*, 653–664, doi:10.1038/s41594-022-00791-x.
265. Sauer, D.B.; Marden, J.J.; Sudar, J.C.; Song, J.; Mulligan, C.; Wang, D.-N. Structural Basis of Ion – Substrate Coupling in the Na+-Dependent Dicarboxylate Transporter VcINDY. *Nat Commun* **2022**, *13*, 2644, doi:10.1038/s41467-022-30406-4.
266. Yu, L.; He, L.; Gan, B.; Ti, R.; Xiao, Q.; Yang, X.; Hu, H.; Zhu, L.; Wang, S.; Ren, R. Structural Insights into Sphingosine-1-Phosphate Receptor Activation. *Proc. Natl. Acad. Sci. U.S.A.* **2022**, *119*, e2117716119, doi:10.1073/pnas.2117716119.
267. Kobayashi, K.; Kawakami, K.; Kusakizako, T.; Miyauchi, H.; Tomita, A.; Kobayashi, K.; Shihoya, W.; Yamashita, K.; Nishizawa, T.; Kato, H.E.; et al. Endogenous Ligand Recognition and Structural Transition of a Human PTH Receptor. *Molecular Cell* **2022**, *82*, 3468-3483.e5, doi:10.1016/j.molcel.2022.07.003.
268. Huang, G.; Liu, D.; Wang, W.; Wu, Q.; Chen, J.; Pan, X.; Shen, H.; Yan, N. High-Resolution Structures of Human Nav1.7 Reveal Gating Modulation through α-π Helical Transition of S6IV. *Cell Reports* **2022**, *39*, 110735, doi:10.1016/j.celrep.2022.110735.
269. Zhao, C.; Xie, Y.; Xu, L.; Ye, F.; Xu, X.; Yang, W.; Yang, F.; Guo, J. Structures of a Mammalian TRPM8 in Closed State. *Nat Commun* **2022**, *13*, 3113, doi:10.1038/s41467-022-30919-y.
270. Young, V.C.; Nakanishi, H.; Meyer, D.J.; Nishizawa, T.; Oshima, A.; Artigas, P.; Abe, K. Structure and Function of H+/K+ Pump Mutants Reveal Na+/K+ Pump Mechanisms. *Nat Commun* **2022**, *13*, 5270, doi:10.1038/s41467-022-32793-0.
271. Zhang, J.; Shi, Y.; Fan, J.; Chen, H.; Xia, Z.; Huang, B.; Jiang, J.; Gong, J.; Huang, Z.; Jiang, D. N-Type Fast Inactivation of a Eukaryotic Voltage-Gated Sodium Channel. *Nat Commun* **2022**, *13*, 2713, doi:10.1038/s41467-022-30400-w.
272. Lin, H.; Xiao, P.; Bu, R.-Q.; Guo, S.; Yang, Z.; Yuan, D.; Zhu, Z.-L.; Zhang, C.-X.; He, Q.-T.; Zhang, C.; et al. Structures of the ADGRG2–Gs Complex in Apo and Ligand-Bound Forms. *Nat Chem Biol* **2022**, *18*, 1196–1203, doi:10.1038/s41589-022-01084-6.
273. Zhu, L.; Zeng, J.; Wang, J. Structural Basis of the Immunity Mechanisms of Pediocin-like Bacteriocins. *Appl Environ Microbiol* **2022**, *88*, e00481-22, doi:10.1128/aem.00481-22.
274. Huang, G.; Wu, Q.; Li, Z.; Jin, X.; Huang, X.; Wu, T.; Pan, X.; Yan, N. Unwinding and Spiral Sliding of S4 and Domain Rotation of VSD during the Electromechanical Coupling in Na $_V$ 1.7. *Proc. Natl. Acad. Sci. U.S.A.* **2022**, *119*, e2209164119, doi:10.1073/pnas.2209164119.
275. Suzuki, S.; Iida, M.; Hiroaki, Y.; Tanaka, K.; Kawamoto, A.; Kato, T.; Oshima, A. Structural Insight into the Activation Mechanism of MrgD with Heterotrimeric Gi-Protein Revealed by Cryo-EM. *Commun Biol* **2022**, *5*, 707, doi:10.1038/s42003-022-03668-3.
276. Killer, M.; Finocchio, G.; Mertens, H.D.T.; Svergun, D.I.; Pardon, E.; Steyaert, J.; Löw, C. Cryo-EM Structure of an Atypical Proton-Coupled Peptide Transporter: Di- and Tripeptide Permease C. *Front. Mol. Biosci.* **2022**, *9*, 917725, doi:10.3389/fmolb.2022.917725.
277. Qian, P.; Nguyen-Phan, C.T.; Gardiner, A.T.; Croll, T.I.; Roszak, A.W.; Southall, J.; Jackson, P.J.; Vasilev, C.; Castro-Hartmann, P.; Sader, K.; et al. Cryo-EM Structures of Light-Harvesting 2 Complexes from *Rhodopseudomonas Palustris* Reveal the Molecular Origin of Absorption Tuning. *Proc. Natl. Acad. Sci. U.S.A.* **2022**, *119*, e2210109119, doi:10.1073/pnas.2210109119.
278. Lam, A.K.M.; Rutz, S.; Dutzler, R. Inhibition Mechanism of the Chloride Channel TMEM16A by the Pore Blocker 1PBC. *Nat Commun* **2022**, *13*, 2798, doi:10.1038/s41467-022-30479-1.
279. Liu, H.; Irobalieva, R.N.; Bang-Sørensen, R.; Nosol, K.; Mukherjee, S.; Agrawal, P.; Stieger, B.; Kossiakoff, A.A.; Locher, K.P. Structure of Human NTCP Reveals the Basis of Recognition and Sodium-Driven Transport of Bile Salts into the Liver. *Cell Res* **2022**, *32*, 773–776, doi:10.1038/s41422-022-00680-4.

280. Zhao, C.; MacKinnon, R. Structural and Functional Analyses of a GPCR-Inhibited Ion Channel TRPM3. *Neuron* **2023**, *111*, 81-91.e7, doi:10.1016/j.neuron.2022.10.002.
281. Lyu, M.; Ayala, J.C.; Chirakos, I.; Su, C.-C.; Shafer, W.M.; Yu, E.W. Structural Basis of Peptide-Based Antimicrobial Inhibition of a Resistance-Nodulation-Cell Division Multidrug Efflux Pump. *Microbiol Spectr* **2022**, *10*, e02990-22, doi:10.1128/spectrum.02990-22.
282. Liu, Y.; Cao, C.; Huang, X.-P.; Gumpper, R.H.; Rachman, M.M.; Shih, S.-L.; Krumm, B.E.; Zhang, S.; Shoichet, B.K.; Fay, J.F.; et al. Ligand Recognition and Allosteric Modulation of the Human MRGPRX1 Receptor. *Nat Chem Biol* **2023**, *19*, 416–422, doi:10.1038/s41589-022-01173-6.
283. Liang, Y.; Plourde, A.; Bueler, S.A.; Liu, J.; Brzezinski, P.; Vahidi, S.; Rubinstein, J.L. Structure of Mycobacterial Respiratory Complex I. *Proc. Natl. Acad. Sci. U.S.A.* **2023**, *120*, e2214949120, doi:10.1073/pnas.2214949120.
284. Wang, H.; Bueler, S.A.; Rubinstein, J.L. Structural Basis of V-ATPase V $_O$ Region Assembly by Vma12p, 21p, and 22p. *Proc. Natl. Acad. Sci. U.S.A.* **2023**, *120*, e2217181120, doi:10.1073/pnas.2217181120.
285. Davies, J.S.; Currie, M.J.; North, R.A.; Scalise, M.; Wright, J.D.; Copping, J.M.; Remus, D.M.; Gulati, A.; Morado, D.R.; Jamieson, S.A.; et al. Structure and Mechanism of a Tripartite ATP-Independent Periplasmic TRAP Transporter. *Nat Commun* **2023**, *14*, 1120, doi:10.1038/s41467-023-36590-1.
286. Jin, Z.; Wan, L.; Zhang, Y.; Li, X.; Cao, Y.; Liu, H.; Fan, S.; Cao, D.; Wang, Z.; Li, X.; et al. Structure of a TOC-TIC Supercomplex Spanning Two Chloroplast Envelope Membranes. *Cell* **2022**, *185*, 4788-4800.e13, doi:10.1016/j.cell.2022.10.030.
287. Yamagata, A.; Murata, Y.; Namba, K.; Terada, T.; Fukai, S.; Shirouzu, M. Uptake Mechanism of Iron-Phytosiderophore from the Soil Based on the Structure of Yellow Stripe Transporter. *Nat Commun* **2022**, *13*, 7180, doi:10.1038/s41467-022-34930-1.
288. Ji, S.-Y.; Dong, Y.-J.; Chen, L.-N.; Zang, S.-K.; Wang, J.; Shen, D.-D.; Guo, J.; Qin, J.; Zhang, H.; Wang, W.-W.; et al. Molecular Basis for the Activation of Thyrotropin-Releasing Hormone Receptor. *Cell Discov* **2022**, *8*, 116, doi:10.1038/s41421-022-00477-0.
289. Tani, K.; Kanno, R.; Kurosawa, K.; Takaichi, S.; Nagashima, K.V.P.; Hall, M.; Yu, L.-J.; Kimura, Y.; Madigan, M.T.; Mizoguchi, A.; et al. An LH1–RC Photocomplex from an Extremophilic Phototroph Provides Insight into Origins of Two Photosynthesis Proteins. *Commun Biol* **2022**, *5*, 1197, doi:10.1038/s42003-022-04174-2.
290. Guo, X.; Wang, Y.; Zhou, J.; Jin, C.; Wang, J.; Jia, B.; Jing, D.; Yan, C.; Lei, J.; Zhou, R.; et al. Molecular Basis for Isoform-Selective Inhibition of Presenilin-1 by MRK-560. *Nat Commun* **2022**, *13*, 6299, doi:10.1038/s41467-022-33817-5.
291. Nan, J.; Yuan, Y.; Yang, X.; Shan, Z.; Liu, H.; Wei, F.; Zhang, W.; Zhang, Y. Cryo-EM Structure of the Human Sodium-Chloride Cotransporter NCC. *Sci. Adv.* **2022**, *8*, eadd7176, doi:10.1126/sciadv.add7176.
292. Laube, E.; Meier-Credo, J.; Langer, J.D.; Kühlbrandt, W. Conformational Changes in Mitochondrial Complex I of the Thermophilic Eukaryote *Chaetomium Thermophilum*. *Sci. Adv.* **2022**, *8*, eadc9952, doi:10.1126/sciadv.adc9952.
293. Ramírez, A.S.; De Capitani, M.; Pesciullesi, G.; Kowal, J.; Bloch, J.S.; Irobalieva, R.N.; Reymond, J.-L.; Aebi, M.; Locher, K.P. Molecular Basis for Glycan Recognition and Reaction Priming of Eukaryotic Oligosaccharyltransferase. *Nat Commun* **2022**, *13*, 7296, doi:10.1038/s41467-022-35067-x.
294. Su, M.; Paknejad, N.; Zhu, L.; Wang, J.; Do, H.N.; Miao, Y.; Liu, W.; Hite, R.K.; Huang, X.-Y. Structures of B1-Adrenergic Receptor in Complex with Gs and Ligands of Different Efficacies. *Nat Commun* **2022**, *13*, 4095, doi:10.1038/s41467-022-31823-1.
295. Wilbon, A.S.; Shen, J.; Ruchala, P.; Zhou, M.; Pan, Y. Structural Basis of Ferroportin Inhibition by Minihepcidin PR73. *PLoS Biol* **2023**, *21*, e3001936, doi:10.1371/journal.pbio.3001936.

296. Yao, X.; Gao, S.; Wang, J.; Li, Z.; Huang, J.; Wang, Y.; Wang, Z.; Chen, J.; Fan, X.; Wang, W.; et al. Structural Basis for the Severe Adverse Interaction of Sofosbuvir and Amiodarone on L-Type Cav Channels. *Cell* **2022**, *185*, 4801-4810.e13, doi:10.1016/j.cell.2022.10.024.
297. Gisriel, C.J.; Shen, G.; Flesher, D.A.; Kurashov, V.; Golbeck, J.H.; Brudvig, G.W.; Amin, M.; Bryant, D.A. Structure of a Dimeric Photosystem II Complex from a Cyanobacterium Acclimated to Far-Red Light. *Journal of Biological Chemistry* **2023**, *299*, 102815, doi:10.1016/j.jbc.2022.102815.
298. Zhang, Z.; Morgan, C.E.; Cui, M.; Yu, E.W. Cryo-EM Structures of AcrD Illuminate a Mechanism for Capturing Aminoglycosides from Its Central Cavity. *mBio* **2023**, *14*, e03383-22, doi:10.1128/mbio.03383-22.
299. Zhao, L.; Yuan, Q.; Dai, A.; He, X.; Chen, C.; Zhang, C.; Xu, Y.; Zhou, Y.; Wang, M.; Yang, D.; et al. Molecular Recognition of Two Endogenous Hormones by the Human Parathyroid Hormone Receptor-1. *Acta Pharmacol Sin* **2023**, *44*, 1227–1237, doi:10.1038/s41401-022-01032-z.
300. Li, J.; Hamaoka, N.; Makino, F.; Kawamoto, A.; Lin, Y.; Rögner, M.; Nowaczyk, M.M.; Lee, Y.-H.; Namba, K.; Gerle, C.; et al. Structure of Cyanobacterial Photosystem I Complexed with Ferredoxin at 1.97 Å Resolution. *Commun Biol* **2022**, *5*, 951, doi:10.1038/s42003-022-03926-4.
301. Lu, M.; Zhao, W.; Han, S.; Lin, X.; Xu, T.; Tan, Q.; Wang, M.; Yi, C.; Chu, X.; Yang, W.; et al. Activation of the Human Chemokine Receptor CX3CR1 Regulated by Cholesterol. *Sci. Adv.* **2022**, *8*, eabn8048, doi:10.1126/sciadv.abn8048.
302. Chen, Z.-P.; Xu, D.; Wang, L.; Mao, Y.-X.; Li, Y.; Cheng, M.-T.; Zhou, C.-Z.; Hou, W.-T.; Chen, Y. Structural Basis of Substrate Recognition and Translocation by Human Very Long-Chain Fatty Acid Transporter ABCD1. *Nat Commun* **2022**, *13*, 3299, doi:10.1038/s41467-022-30974-5.
